# Peanut oral immunotherapy differentially suppresses clonally distinct subsets of T helper cells

**DOI:** 10.1172/JCI150634

**Published:** 2022-01-18

**Authors:** Brinda Monian, Ang A. Tu, Bert Ruiter, Duncan M. Morgan, Patrick M. Petrossian, Neal P. Smith, Todd M. Gierahn, Julia H. Ginder, Wayne G. Shreffler, J. Christopher Love

**Affiliations:** 1 Koch Institute for Integrative Cancer Research,; 2 Department of Chemical Engineering, and; 3 Department of Biological Engineering, Massachusetts Institute of Technology (MIT), Cambridge, Massachusetts, USA.; 4 Center for Immunology and Inflammatory Diseases, Massachusetts General Hospital (MGH), Boston, Massachusetts, USA.; 5 Harvard Medical School, Boston, Massachusetts, USA.; 6 Broad Institute of MIT and Harvard, Cambridge, Massachusetts, USA.

**Keywords:** Immunology, Allergy, T cells

## Abstract

Food allergy affects an estimated 8% of children in the United States. Oral immunotherapy (OIT) is a recently approved treatment, with outcomes ranging from sustained tolerance to food allergens to no apparent benefit. The immunological underpinnings that influence clinical outcomes of OIT remain largely unresolved. Using single-cell RNA-Seq and paired T cell receptor **α**/**β** (TCR**α**/**β**) sequencing, we assessed the transcriptomes of CD154^+^ and CD137^+^ peanut-reactive T helper (Th) cells from 12 patients with peanut allergy longitudinally throughout OIT. We observed expanded populations of cells expressing Th1, Th2, and Th17 signatures that further separated into 6 clonally distinct subsets. Four of these subsets demonstrated a convergence of TCR sequences, suggesting antigen-driven T cell fates. Over the course of OIT, we observed suppression of Th2 and Th1 gene signatures in effector clonotypes but not T follicular helper–like (Tfh-like) clonotypes. Positive outcomes were associated with stronger suppression of Th2 signatures in Th2A-like cells, while treatment failure was associated with the expression of baseline inflammatory gene signatures that were present in Th1 and Th17 cell populations and unmodulated by OIT. These results demonstrate that differential clinical responses to OIT are associated with both preexisting characteristics of peanut-reactive CD4^+^ T cells and suppression of a subset of Th2 cells.

## Graphical abstract

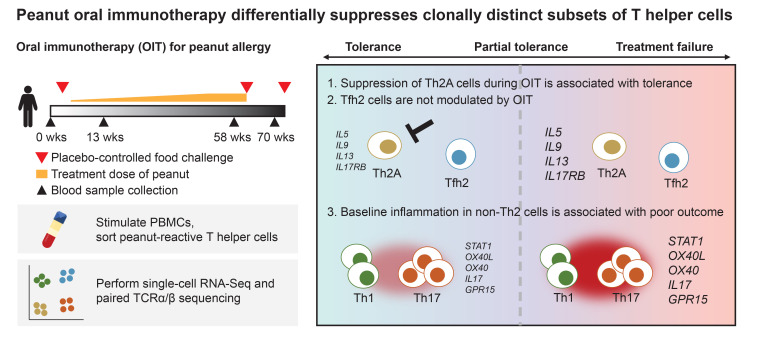

## Introduction

Food allergy is an immune hypersensitivity condition characterized by high-affinity allergen-specific IgE antibodies and allergen-specific T helper 2 (Th2) cells (1–3). Specific IgE binds to effector cells, such as mast cells and basophils, through FcεRI receptors that are cross-linked upon binding of allergen. The resulting cellular degranulation causes local and systemic release of histamine and other mediators, leading to allergic reactions ranging from mild symptoms, such as hives and abdominal pain, to potentially life-threatening anaphylaxis (4). Allergen-specific Th2 cells constitute a critical component in this cascade. Th2 cells are broadly defined by expression of the transcription factor GATA3 and secretion of the cytokines IL-4, IL-5, and IL-13, which promote class-switching of B cells to IgE and the recruitment of other effector cells, such as eosinophils (5, 6). Recent studies have highlighted subtypes of Th2 cells with specialized functions in the context of allergy, including effector memory (e.g., Th2A, pathogenic effector Th2 [peTh2]), and T follicular helper (e.g., Tfh13) phenotypes (6–10).

Oral immunotherapy (OIT) is currently the only FDA-approved treatment for food allergy intended to prevent anaphylaxis (11). OIT involves the daily ingestion of escalating doses of allergen. Most patients (80%–85%) achieve desensitization (a loss in clinical reactivity with regular consumption of the allergen), but only about one-third of patients maintain unresponsiveness if treatment is discontinued for even just a few months (12–14). Studies of the impact of OIT on circulating T cells have consistently found evidence for suppression of Th2 responses, but most of these studies have not correlated T cell responses with heterogenous clinical outcomes (7, 15–18). Similarly, while Treg induction has been observed using in vitro expansion of T cells from patients undergoing OIT, it has not been consistently shown ex vivo (17–24). Studying allergen-reactive T cell subsets ex vivo is challenging because of their low frequencies in peripheral blood and technical constraints, which limit the ability to reliably phenotype these populations and longitudinally track corresponding clonotypes (22, 25). As a result, existing data on T cell responses in the context of OIT have been limited to features comprising a narrow set of genes and proteins, or T cells specific for a predefined subset of allergen epitopes (17, 22). Comprehensive characterization of allergen-specific CD4^+^ T cell subsets and their response to immunotherapy over time may not only refine strategies for the treatment of food allergy, but may also enhance our broader understanding of Th cell phenotypes in atopic disease.

## Results

### Single-cell RNA-Seq enables deep profiling of peanut-reactive Th cells from OIT patients.

**Figure 1:**
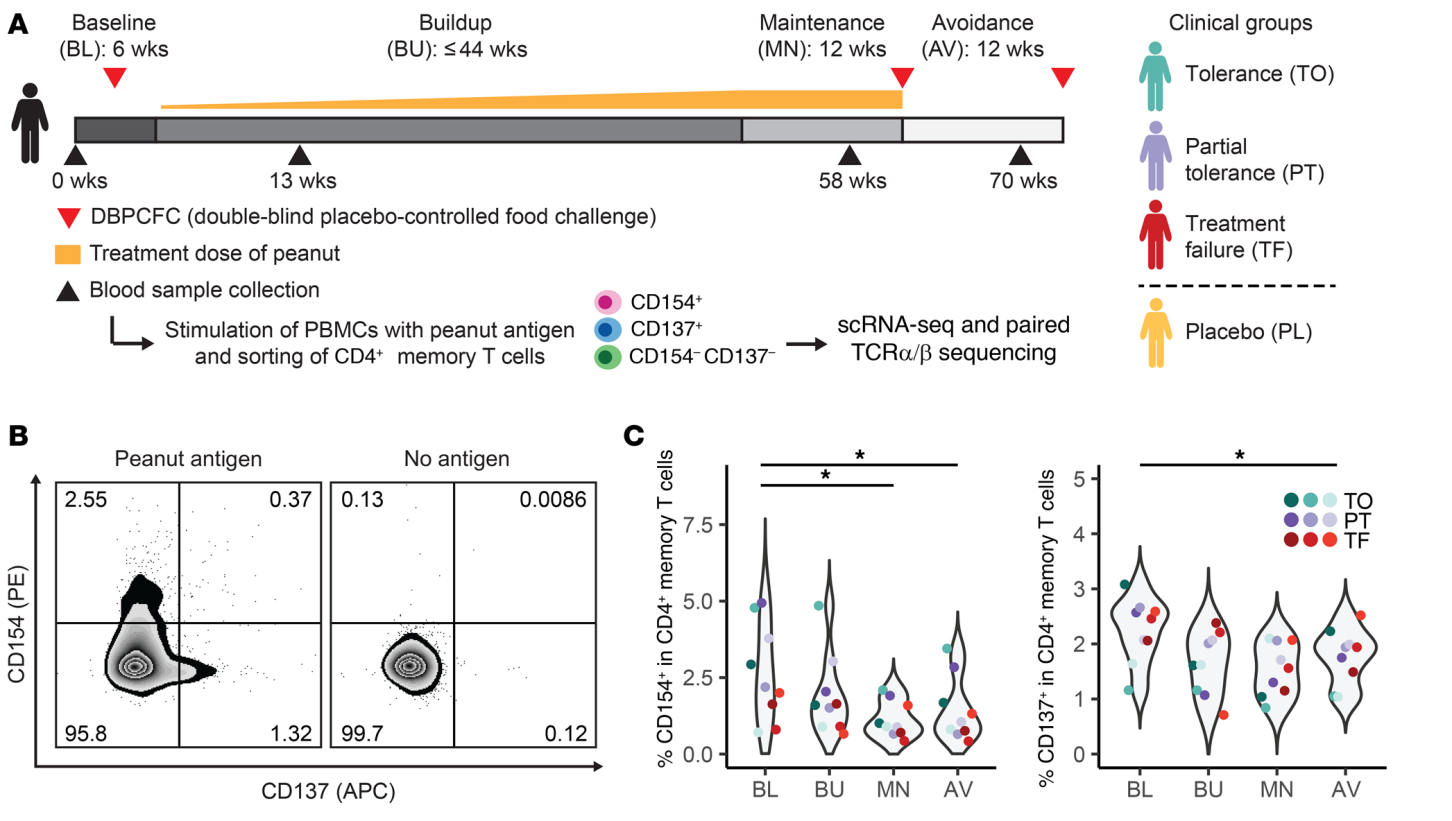
Peanut-reactive T cells decrease in frequency over the course of OIT. (**A**) OIT study design, sample processing, and patient cohorts. CD3^+^CD4^+^CD45RA^–^ memory T cells were sorted by FACS as CD154^+^CD137^+/–^ (CD154^+^), CD154^–^CD137^+^ (CD137^+^), or CD154^–^CD137^–^. (For clinical outcomes and patient information, see Methods and Supplemental Tables 1 and 2.) (**B**) Representative flow plots of cells from 1 patient at 1 time point (*n =* 12 patients total). (**C**) Percentage of CD4^+^ memory T cells at each time point that were CD154^+^ (left) or CD137^+^ (right) in peanut-stimulated PBMC cultures from patients in the treatment group. *P < 0.05 (adjusted), by paired Wilcoxon rank sum test. AV, avoidance; BL, baseline; BU, buildup; MN, maintenance; PL, placebo; PT, partial tolerance; TF, treatment failure; TO, tolerance.

To measure the impact of OIT on peanut-reactive T cells, we profiled longitudinal blood samples from 12 patients participating in a clinical trial of peanut OIT (ClinicalTrials.gov identifier, NCT01750879; Supplemental Tables 1 and 2; supplemental material available online with this article; https://doi.org/10.1172/JCI150634DS1). In brief, we isolated PBMCs from each patient at 4 time points: baseline (before therapy), buildup (13 weeks after the start of therapy), maintenance (12 weeks after the maximum dose was reached), and avoidance (12 weeks after the end of therapy). Clinical outcomes were evaluated by 2 oral food challenges and were defined as: tolerance (passing both food challenges); partial tolerance (passing the challenge at the maintenance time point but failing the challenge at the avoidance time point); and treatment failure (failing the challenge at the maintenance time point). Samples from 3 patients treated with placebo were also included (Figure 1A and see Methods). Consistent with prior studies, peanut-specific IgE levels showed a transient increase at buildup (20, 26); however, peanut-specific IgE concentrations did not correlate with clinical outcomes at any time point (Supplemental Figure 1).

To enrich for allergen-specific T cells and capture their activated profiles, we cultured the PBMCs with whole peanut protein extract for 20 hours to activate CD4^+^ memory T cells. Peanut-reactive cells were then enriched via FACS using CD154 and CD137 (activation markers for effector and regulatory T cell states, respectively) (Figure 1, A and B, Supplemental Figure 2A, and refs. 27–29). This approach allowed us to recover a broad set of peanut-specific T cells with limited bias for specific epitopes or HLA types (29). The 20-hour stimulation duration was intended to capture ex vivo cell states and reflect in vivo clonal distributions; it provides sufficient time for the processing of whole peanut proteins by antigen-presenting cells and the activation of peanut-reactive CD4^+^ T cells, but it is too short to induce substantial proliferation of antigen-activated T cells (27, 28, 30, 31). CD154-based approaches have been broadly used to identify antigen-reactive CD4^+^ T cells in various contexts (25, 32–34). In addition, we have previously shown that the frequency of peanut-reactive CD154^+^CD4^+^ T cells in patients with peanut allergy is correlated with the patients’ clinical sensitivity, illustrating the specificity of this assay (10, 35). Using this method, we observed that OIT significantly decreased the frequency of peanut-reactive CD154^+^ and CD137^+^ T cells in the peripheral blood (Figure 1C); this trend was not observed in the placebo group (Supplemental Figure 2B). The frequency of CD154^+^ T cells in unstimulated cultures from the same patients was low, indicating that CD154 expression was induced by peanut stimulation and not associated with activated memory T cells already present in the peripheral blood (Supplemental Figure 2C).

To further characterize peanut-reactive memory CD4^+^ T cells and study how their phenotypes and repertoire are altered during OIT in relation to treatment outcome, we processed the sorted cells for single-cell RNA-Seq via Seq-Well and paired single-cell T cell receptor α/β (TCRα/β) sequencing (36, 37). We also processed CD154^–^CD137^–^ cells from a subset of patients for use as controls. After filtering cell transcriptomes for library quality, we recovered high-quality transcriptomes for 134,129 cells (see Methods and Supplemental Figure 3A).

**Figure 2:**
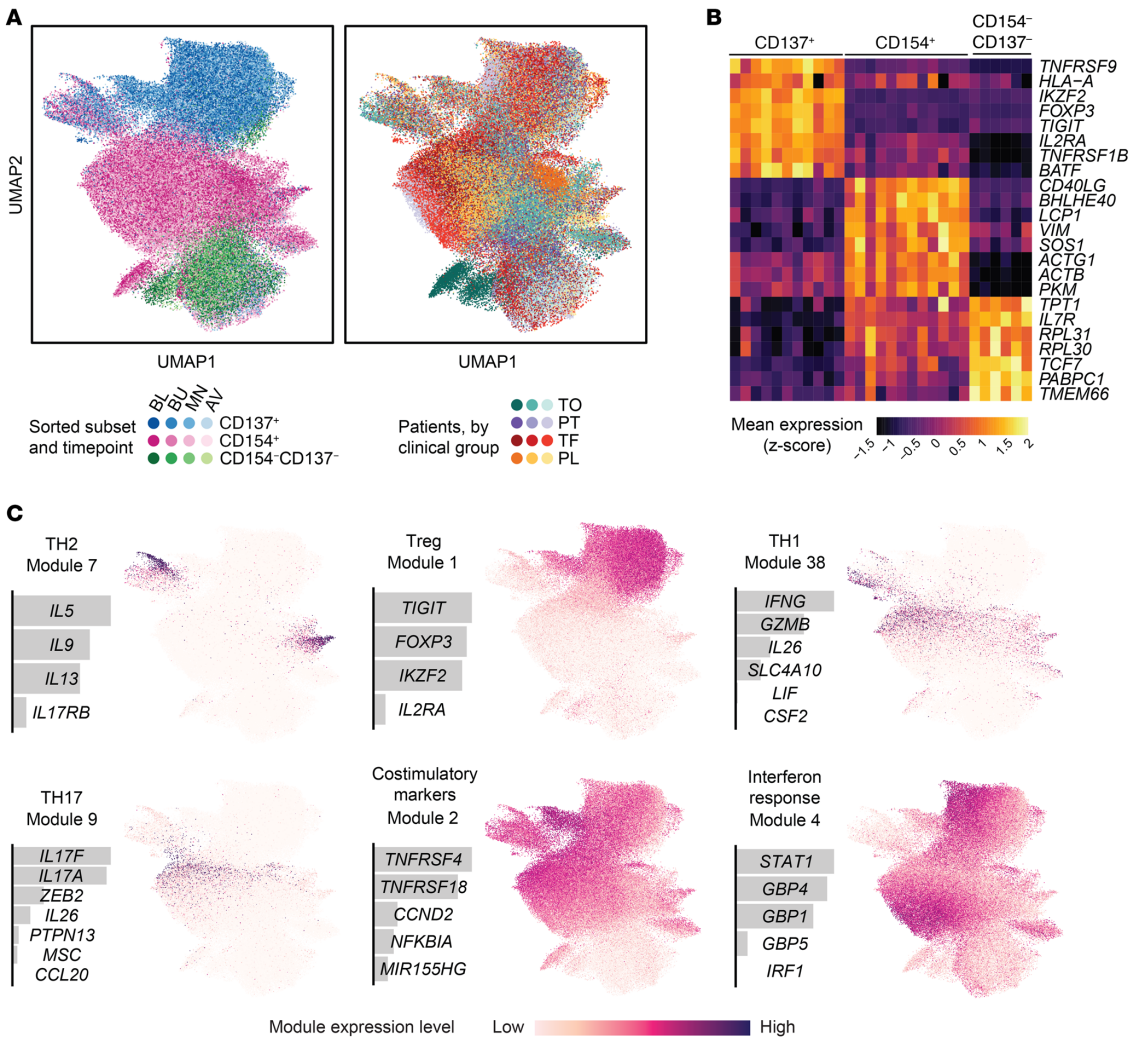
Peanut-reactive T cells from patients undergoing OIT have diverse and distinct transcriptional signatures. (**A**) 2D UMAP visualization of all single-cell transcriptomes (*n =* 134,129 cells), colored by sorted subset and time point (left) and by patient and clinical group (right). (**B**) Top differentially expressed genes between the sorted subsets. Each column represents the scaled average gene expression of cells from a single patient. Genes were selected using a receiver operating characteristic (ROC) test. (**C**) Selected gene modules discovered using sparse PCA, labeled with module number and a proposed descriptor. For each module, the relative weights of each contributing gene and the module score of all cells overlaid on the UMAP coordinates are shown.

Peanut-reactive T cell transcriptomes formed clusters associated most closely with their sorted subsets (Figure 2A). We observed patient-specific variation within each cluster that was not a function of library size or mitochondrial content (Figure 2B and Supplemental Figure 3), suggesting that it represented inherent biological rather than technical differences. CD154^+^ and CD137^+^ cells were separated by many differentially expressed genes, including their associated transcripts *CD40LG* and *TNFRSF9*, the Treg marker *FOXP3*, and others consistent with effector and regulatory phenotypes, respectively (Figure 2C). Qualitatively, there was no strong association between transcriptome (as measured by uniform manifold approximation and projection [UMAP] embeddings) and time point or treatment outcome, suggesting that OIT-induced effects might be subtle rather than dominant in the data.

### Sparse principal component analysis delineates canonical and new Th cell gene modules.

To uncover evidence of OIT-driven variation among peanut-reactive T cells, we developed an unsupervised approach to identify conserved programs of immune-related gene expression. The data set was filtered to 937 immune and variable genes (Supplemental Table 3). Then, coexpressed genes were aggregated into gene modules using sparse principal component analysis (PCA) (38) to derive a set of 50 gene modules (see Methods and Supplemental Figures 4 and 5). Several modules corresponded with phenotypes of known T cell subsets, such as Th1, Th2, Th17, and Tregs (Figure 2C). Forty-three of 50 gene modules were present across most or all of the patients (Supplemental Figure 6 and see Methods), indicating that these represent programs of T cell function or activation that are consistent among individuals.

### Th-related gene modules are associated with expanded T cells.

**Figure 3:**
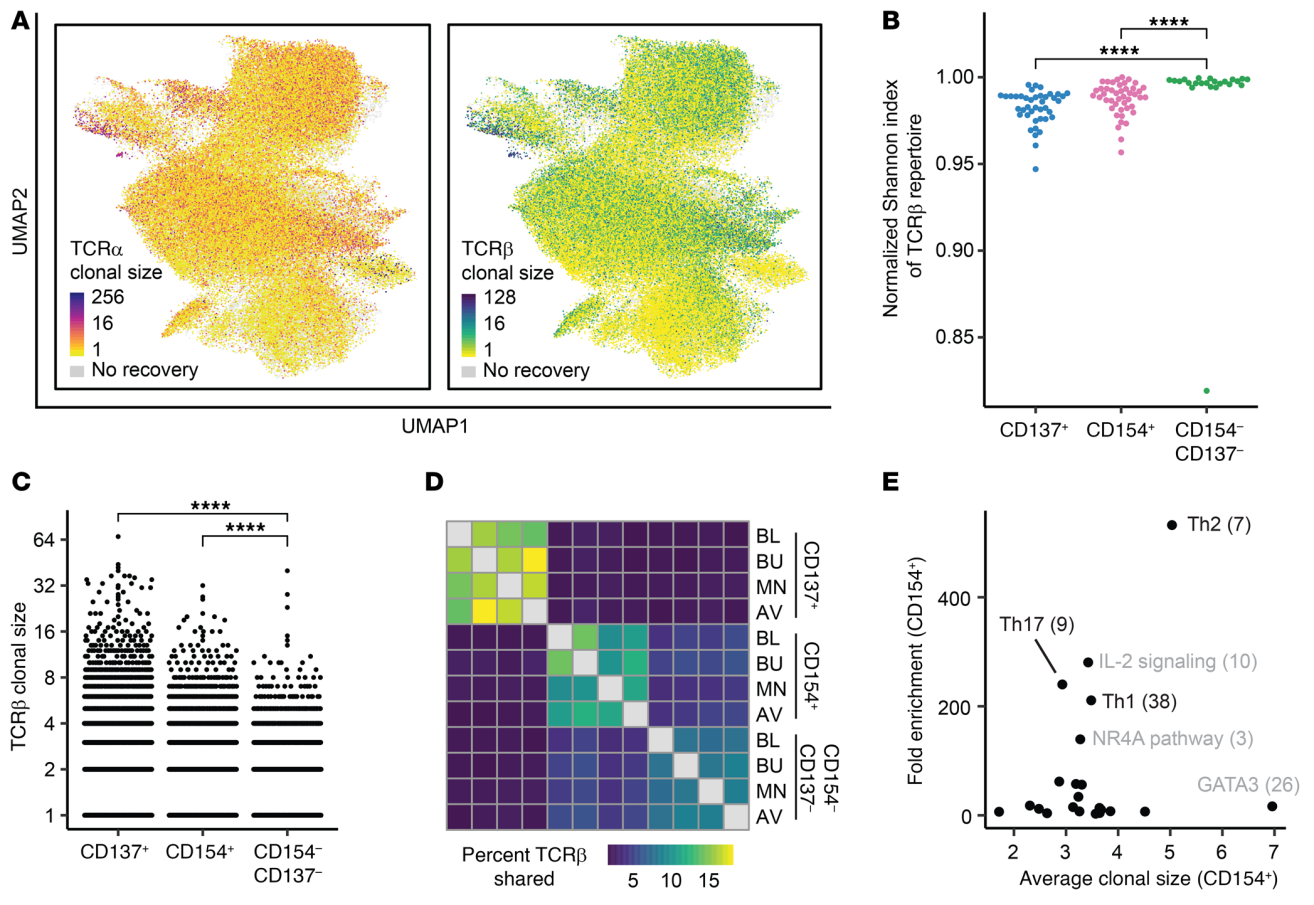
Gene modules for Th function are associated with clonal expansion and expression in activated cells. (**A**) Clonal size of TCRα sequence (left) or TCRβ sequence (right) for all cells with paired TCR recovery, overlaid onto UMAP coordinates. Clonal size is defined as the number of cells sharing a TCR sequence. (**B**) Diversity (normalized Shannon index) of TCRβ repertoires of each sorted subset. Each data point represents the repertoire for 1 patient at 1 time point (CD137^+^: *n =* 41; CD154^+^: *n =* 44; CD154^–^CD137^–^: *n =* 23). (**C**) Distribution of TCRβ clonal sizes, within each sorted subset. Cells within each sorted subset were downsampled to equal numbers before clonal sizes were calculated. (**D**) Percentage of TCRβ sequences shared between time points and sorted subsets. The percentage shared is defined as the number of unique TCRβ sequences detected in both conditions, divided by the geometric mean of the number of unique TCRβ sequences in each of the 2 conditions. Sequences from all patients with samples in all 3 conditions (*n =* 6 patients) were pooled. (**E**) Mean clonal size and fold change in mean module scores (compared with module-expressing CD154^–^CD137^–^ cells) in CD154^+^ cells expressing each gene module. Each data point represents a single gene module. Cells were classified as “expressing” each module or not, relative to background expression (see Methods). Clonal size was calculated with respect to all cells in the data set. ****P < 0.0001 (adjusted), by unpaired Wilcoxon rank-sum test. Data represent combined data from all patients at all time points (**A**–**C** and **E**).

To investigate clonal T cell responses to peanut antigens, we recovered paired TCR sequences from a single-cell, whole-transcriptome amplification product. We identified TCRβ sequences for 60% (±17%), TCRα for 55% (±15%), and both chains for 36% of cells (±12%) (numbers represent the median ± SD across patients). Coverage was uniform across samples, and the majority of expanded TCRβ sequences were paired with a single TCRα (Figure 3A and Supplemental Figure 7). Given this relationship, we used TCRβ for all subsequent analyses involving clonotypes. The diversities of CD154^+^ and CD137^+^ repertoires were significantly lower than those of the CD154^–^CD137^–^ cells, indicating that these activation markers enriched for a pool of clonally expanded, peanut-reactive clonotypes (Figure 3, B and C). In addition, we observed that 55% of expanded clones were detected across multiple time points, but only 1.6% of clonotypes were shared between CD154^+^ and CD137^+^ cells, suggesting that these 2 activated subsets resulted from fundamental differences in lineage, epitope specificity, or both (Figure 3D).

To determine which, if any, gene modules were associated with clonal T cell expansion, we classified cells as expressing or nonexpressing for each module, on the basis of whether the module score was above background expression in CD154^–^CD137^–^ cells (see Methods). We then calculated the average TCRβ clonal size for cells expressing each module, as well as the average score of that module in CD154^+^ cells relative to CD154^–^CD137^–^ cells. We found that modules representing Th1, Th2, and Th17 functions exhibited strong upregulation in both the CD154^+^ and CD137^+^ compartments and were associated with expanded T cell clonotypes, suggesting that these phenotypes were largely associated with peanut-reactive clonotypes rather than with bystander-activated, non-peanut-reactive T cells (Figure 3E and Supplemental Figure 8).

### Peanut-reactive Th cells include 6 phenotypically distinct states.

**Figure 4:**
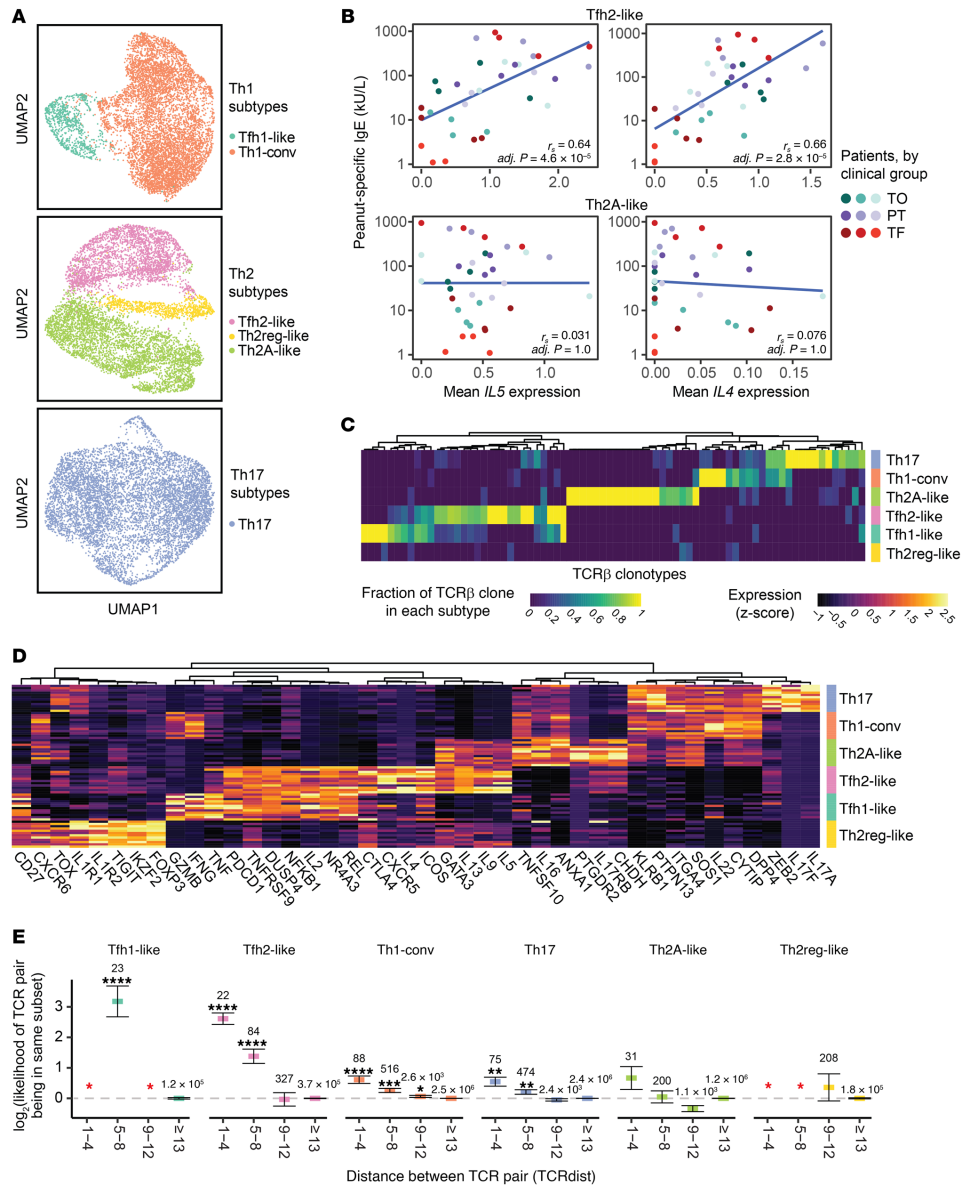
Peanut-reactive Th subtypes are clonally distinct and exhibit TCR convergence. (**A**) UMAP visualizations of Th1- (*n =* 7,609 cells), Th2- (*n =* 7,877 cells), and Th17-scoring cells (*n =* 7111 cells). Clusters are annotated by their putative identity. (**B**) Scatter plots of the average expression of *IL5* and *IL4* in Tfh2-like cells or Th2A-like cells (for each patient at each time point) and peanut-specific IgE titers. Linear fit, Spearman’s correlation (*rs*, *n =* 34), and adjusted *P* values are shown. (**C**) Fraction of TCRβ clonotypes belonging to each subset. The fraction is defined as the number of cells of a TCRβ CDR3 sequence (column) detected in each Th subset, divided by the total number of cells within the clonotype. Clonotypes were randomly downsampled to visualize a comparable number from each subset. (**D**) Differentially expressed genes in each Th subset. Genes were selected using an ROC test and manual curation. Each row represents the scaled average gene expression in 1 patient. (**E**) TCR distance analysis of TCR sequences. The *x* axis represents bins of increasing pairwise TCR distance, calculated using TCRdist, and the *y* axis represents the likelihood of pairs of cells at a given TCR distance to be of the same Th subset, normalized to the prior probability of any 2 cells belonging to that subset (see Methods). ****P < 0.0001 (adjusted), ***P < 0.001 (adjusted), and **P < 0.01 (adjusted), by 2-sided χ^2^ proportion test with 1 degree of freedom. The total number of pairs within each TCR distance and subset is indicated above each data point. The red asterisk indicates that no pair of TCR sequences with the respective TCR distance bin was found in the respective subset. Error bars represent 85% binomial CIs. Data were combined from all patients at all time points (**A**–**E**).

Given their strong enrichment in the CD154^+^ and CD137^+^ compartments, we further analyzed the heterogeneity among cells expressing the Th1, Th2, and Th17 modules. Separate clustering of these cells revealed 3 phenotypically distinct clusters of Th2 cells and 2 clusters of Th1 cells. We did not observe additional clusters within the Th17 cells (Figure 4A). These clusters were detected in all patients (Supplemental Figure 9). Within the Th2 cells, the clusters corresponded to a Tfh2-like cell population (high in costimulatory markers, *CXCR5*, and *PDCD1*), a Th2 regulatory–like (Th2reg-like) cell population (*FOXP3* and *TNFRSF9*), and a Th2A-like cell population (7, 39) (*GATA3*, *IL17RB*, and *PTGDR2*) (Figure 4D). The Tfh2-like population resembled a previously described pathogenic Tfh13 subset, whereas the Th2A-like population shared markers previously identified in Th2A and peTh2 populations (refs. 7–9 and Supplemental Figure 10). Likewise, the Th2reg-like population shared features with previously described deviated Tregs in food allergy (40). Among the Th1 cells, the clusters corresponded to a Tfh1-like population and a conventional Th1 (Th1-conv) cell population with canonical Th1 signatures (ref. 41 and Figure 4, A and D). Both of these clusters expressed high levels of *IFNG* and *GZMB*, and the Tfh1-like cluster exhibited a high overlap of genes with those expressed in the Tfh2-like population, including *ICOS*, *PDCD1*, and *TNFRSF9*.

We hypothesized that Tfh2-like cells influence the class-switching of peanut-specific B cells to IgE. To investigate this hypothesis, we determined the correlation between the average expression of each gene expressed by Tfh2-like cells and peanut-specific IgE titers for each patient at each time point. In total, we detected 66 genes that were significantly correlated with peanut-specific IgE levels in plasma (Supplemental Figure 11). Transcripts positively correlated with IgE included the Th2 cytokines *IL5* and *IL4*; this correlation was observed in Tfh2-like, but not Th2A-like, cells (Figure 4B). Other positive correlates with IgE included the costimulatory receptor *ICOS*, the gut-homing integrin *ITGA4*, and *PLA2G16* and *GK*, two transcripts implicated in the production of prostaglandin-D2 by peTh2 cells in eosinophilic esophagitis (42), whereas transcripts negatively correlated with IgE production included *TGFB1*, which is associated with class-switching to IgA (43, 44), and *TNFSF10*, which has been demonstrated to dampen Th2 responses in allergic asthma (ref. 45 and Supplemental Figure 11). No genes expressed by Th2A-like cells were significantly correlated with peanut-specific IgE. These results demonstrate a relationship between gene expression in Tfh2-like cells and peanut-specific IgE levels and suggest that cytokine signals from different Th2 subsets may contribute differently to class-switching to IgE.

### Peanut-reactive Th cell phenotypes are clonally distinct.

We next sought to determine the clonal relationships present among the distinct phenotypes of peanut-reactive T cells. Analysis of the TCR repertoires of the 6 Th subtypes showed that most clones were primarily associated with a single subtype, indicating that these populations represent distinct clonal lineages (Figure 4C). We did, however, observe overlapping clones between the Th1-conv and Th17 states as well as the Tfh1-like and Tfh2-like states, suggesting that cells may transition between these pairs of phenotypic states, or that these states may include shared cellular lineages that differentiated relatively late (46).

To determine to what extent this association between clonotype and phenotype might be influenced by epitope recognition, we next assessed whether TCRs showed evidence of convergence within Th subtypes using TCRdist, a quantitative metric for similarity between a pair of TCR sequences (ref. 47, and see Methods). A pair of cells with very similar TCR sequences may share epitope-binding properties despite having different ancestries, allowing an assessment of the role of epitope recognition in shaping T cell phenotypes. We found that pairs of cells with highly similar TCRβ sequences (TCRdist <9) had a significantly increased likelihood of both cells belonging to the same Th subtype (*P <* 0.05, by χ^2^ proportion test), with the exception of cells in the Th2A-like and Th2reg-like subtypes (Figure 4E). This result indicates a convergence onto common TCR motifs within most subtypes and suggests that factors such as TCR affinity or antigen context during priming (e.g., local tissue environment) may influence the induction of specific Th phenotypes within an individual (48–50).

### OIT suppresses Th2 and Th1 signatures in conventional effector, but not Tfh-like, cells.

**Figure 5:**
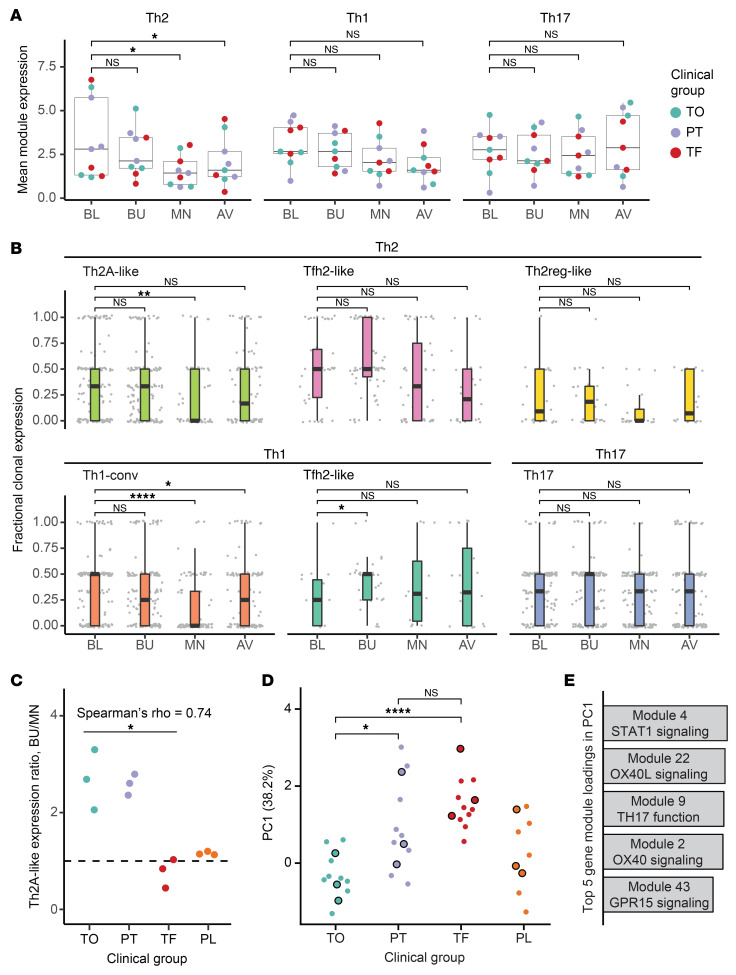
Th1 and Th2 effector, but not Tfh-like, subsets are suppressed by OIT. (**A**) Mean Th2, Th1 and Th17 gene module expression over time within Th2, Th1, and Th17 clones (see Methods), respectively, in each treatment group patient at each time point. (**B**) Fractional expression of Th2, Th1, and Th17 modules within clonotypes of Th subtypes over time. Fractional clonal expression is defined as the proportion of cells within each clonotype expressing their respective module (see Methods). Each data point represents the cells of an individual expanded clonotype from 1 patient at 1 time point. Patients in the placebo group were excluded. (**C**) Degree of suppression in Th2A-like clones by clinical group. The ratio of mean Th2 module expression in Th2A-like clones from each patient was calculated between buildup and maintenance. Spearman’s rho = 0.74; *P < 0.05. *n =* 9. Spearman’s test was performed to determine the correlation between ratio and outcome within the treatment group (assigning 2 for tolerance, 1 for partial tolerance, and 0 for treatment failure to represent the ordinal relationship between treatment groups). (**D**) PC1 score for CD154^+^ cells by outcome. PCA was performed using the 50 gene modules as features and all CD154^+^ cells at baseline as the input data (see Methods). Each data point represents the mean PC1 score for all CD154^+^ cells from a single patient at a single time point. Black-outlined data points represent the baseline time point. (**E**) Top 5 gene module loadings in PC1. Bar heights represent the magnitude of each contribution to PC1. (See Supplemental Figures 4 and 5 for further details on each gene module.) *P < 0.05 (adjusted), **P < 0.005 (adjusted), and ****P < 0.0005 (adjusted), by paired (**A**) or unpaired (**B** and **D**) Wilcoxon rank-sum test.

We next assessed the impact of OIT on the TCR repertoire and the identified Th subtypes. The majority of expanded CD154^+^ and CD137^+^ clonotypes were present at 3 or all 4 of the time points, and no time point was associated with the depletion or emergence of unique expanded clonotypes or singletons, suggesting that OIT did not induce strong changes in the TCR repertoires of peanut-reactive CD154^+^ or CD137^+^ cells from peripheral blood (Supplemental Figure 12). Next, we evaluated phenotypic changes within peanut-reactive Th1, Th2, and Th17 clones during OIT by assessing the mean expression of their respective modules over time in each patient. Each set of Th clones was defined as all clonotypes in which the relevant module (e.g., Th2) was expressed in at least 1 cell at any time point; this definition allowed us to include peanut-reactive T cells that may gain or lose Th gene expression as a result of OIT (see Methods). We found evidence of suppression in Th2 and, to a lesser extent, Th1 clones (adjusted *P* values of 0.036 and 0.117, respectively) between the baseline and maintenance time points (Figure 5A). We did not observe this trend in patients treated with placebo (Supplemental Figure 13B).

To determine which of the previously defined 6 Th subtypes were associated with this Th2 and Th1 suppression, we next assigned each Th1, Th2, and Th17 clonotype to the Th subtype in which it most frequently appeared. We then quantified changes in gene module expression within each individual clonotype, an analysis that allowed us to track the phenotypes of hundreds of individual clonal lineages over the course of treatment. As a way to measure the stability of module expression over time within each clonotype, we calculated its fractional clonal expression: the proportion of cells that expressed the corresponding module (Th2, Th1, or Th17) at each time point (see Methods). From this analysis, we found that Th1-conv and Th2A-like clonotypes exhibited suppression of Th1 and Th2 genes, respectively, at the maintenance time point compared with baseline. This suppression was consistent with an anergic state, characterized by decreased cytokine expression in response to stimulation and was not detected in the placebo group (Supplemental Figure 13C). In contrast, we did not observe statistically significant changes in module expression at the clonotype level in the Tfh1-like, Tfh2-like, Th2reg-like, or Th17 subsets (Figure 5B), suggesting that these cell populations were more refractory to modulation by OIT than Th1-conv and Th2A-like clonotypes. A lack of suppression of Th2A-like clonotypes at maintenance was associated with poor outcomes (Spearman’s rho = 0.74; *P* = 0.02), and the degree of suppression was similar between patients who achieved partial tolerance and full tolerance (Figure 5C). We found no statistically significant association between clinical outcome and degree of suppression in Tfh2-like clonotypes or Th1-conv clonotypes (Supplemental Figure 13A).

### Non-Th2 inflammatory pathways at baseline are associated with clinical outcome.

While a lack of Th2 suppression during OIT was associated with a poor clinical outcome, the baseline expression of Th2 signatures was not predictive. To analyze immune signatures present at the beginning of treatment, we performed PCA on gene module scores of all CD154^+^ cells at baseline. This approach allowed us to assess major axes of phenotypic variation among CD154^+^ cells at baseline and investigate whether any of these axes correlated with clinical outcome. We found a striking separation by outcome at all time points in the scores of the first principal component (PC1) alone, with high PC1 scores associated with poor clinical outcome (Figure 5D). The top gene modules enriched in PC1 were defined by markers of T cell activation and effector response such as OX40, OX40L, Th17 function, STAT1, and GPR15 (Figure 5E and Supplemental Figure 14). To investigate the cell types associated with this signature, we summarized PC1 scores and module expression in the 6 previously identified Th subtypes. Of these, Th1-conv and Th17 cells expressed the highest levels of PC1 (Supplemental Figure 15). Consistent with this observation, the frequencies of Th1-conv and Th17, but not Th2, cells were also lower in the CD154^+^ compartment of patients with favorable clinical outcome (Supplemental Figure 9A). Interestingly, CD154^+^ cells not classified within any of the canonical CD4^+^ T cell subtypes also showed outcome-dependent expression of modules associated with PC1 (Supplemental Figure 16). These results indicate that a range of CD4^+^ T cell phenotypes and inflammatory pathways may affect the likelihood of favorable responses to OIT.

### Treg phenotypes are not significantly modulated by OIT.

**Figure 6:**
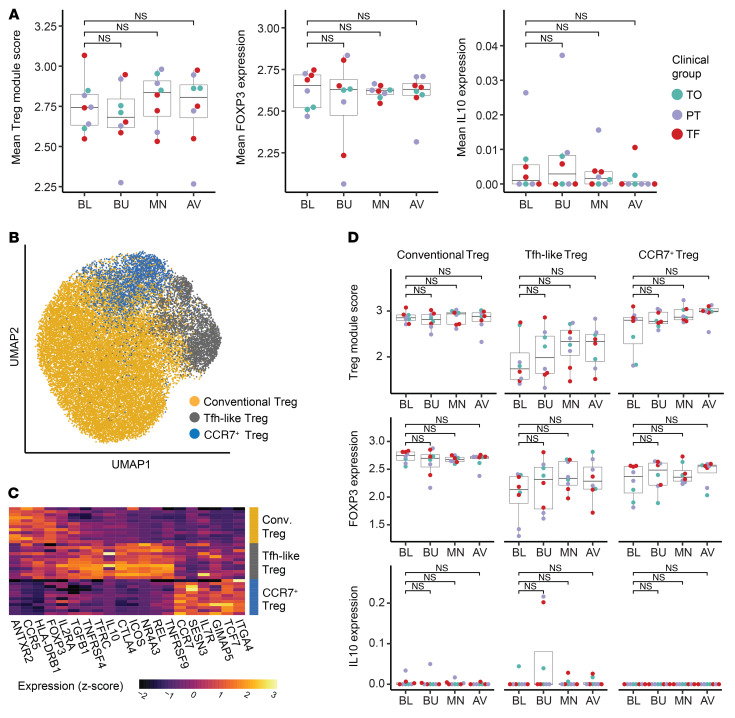
Treg phenotypes are not significantly modulated by OIT. (**A**) Average expression of Treg module (gene module 1), *FOXP3*, and *IL10* by patient and time point within Treg clones. Each data point represents the mean expression of all Treg clones for a given patient at a given time point. (**B**) UMAP visualization of all Tregs (data from all patients at all time points), colored by cluster assignment and labeled by putative cluster identity. (**C**) Differentially expressed genes in each Treg cluster. Genes were selected using an ROC test and manual curation. Each row represents the scaled average gene expression in 1 patient. (**D**) Average expression of the Treg module (top), *FOXP3* (middle), and *IL10* (bottom) by patient and time point within clones of each Treg cluster, colored by clinical group. Adjusted *P* values were calculated by paired Wilcoxon rank sum test (**A** and **D**).

Tregs have been described in some studies as a correlate of favorable clinical outcome in OIT (19, 20). Although we detected a strong and sustained expression of Treg markers among peanut-reactive CD137^+^ cells, we observed a moderate decrease in the frequency of CD137^+^ cells over the course of OIT (Figure 1C). In addition, although *IL10* in Treg module–expressing clones (gene module 1) was slightly elevated during the buildup phase of treatment, there was not a sustained increase in the expression of the Treg module, *FOXP3*, or *IL10* among these cells over OIT (Figure 6A). Moreover, expression of either *IL10* or *FOXP3* did not correlate with clinical outcome. Unsupervised analysis of Treg module–expressing cells revealed 3 distinct subsets of Tregs, including conventional Tregs, Tfh-like Tregs, and CCR7^+^ Tregs, which differed in their expression of *IL10*, *IL2RA*, and several costimulatory and memory markers (Figure 6, B and C). For example, Tfh-like Tregs were responsible for nearly all of the *IL10* expression. No Treg cluster showed a sustained increase of *FOXP3*, *IL10*, or the Treg gene module as a result of OIT (Figure 6D). Finally, we saw no evidence of the induction of new peanut-reactive Treg clonotypes during OIT, as TCR repertoires of CD137^+^ cells remained stable over time (Supplemental Figure 12). Our data indicate a lack of induction of peanut-reactive Tregs during OIT, both by gene expression levels and by clonotype frequencies.

## Discussion

In this study, we characterized peanut-reactive Th cells from allergic patients undergoing OIT using single-cell RNA-Seq with paired TCR sequencing. These methods allowed us to identify patterns of expansion and TCR convergence among distinct peanut-reactive Th subtypes and to longitudinally profile individual clonotypes throughout OIT. We found differential effects of OIT on distinct Th subtypes and a significant association at baseline between T cell phenotypes and clinical outcome. Our results add refinement to the transcriptomic-scale definitions of previously described subsets, reveal how clonotypes from these cell populations are affected during OIT, and provide additional insight into the substantial heterogeneity of patients with peanut allergy.

Among sorted CD154^+^ and CD137^+^ Th cells, we identified 6 subtypes of highly clonal peanut-reactive Th cells with Th1, Th2, and Th17 signatures. Of these subtypes, the Th2A-like, Tfh2-like, and Th2reg-like cells corresponded well to the previously described Th2A, Tfh13, and deviated Treg populations in food allergy (7–9, 40). We show here for the first time to our knowledge that these subsets have distinct TCR repertoires and that some are enriched in highly similar TCR sequences. Our results add resolution to a previous study showing that distinct repertoires exist between CD154^+^ and CD137^+^ cells (29). This segregation of TCR repertoires strongly suggests that the subsets represent distinct lineages rather than transient phenotypes, and the phenomenon of TCR convergence hints at a skewing of the T cell state due to epitope interactions or epitope-associated factors (50–52). We did not detect significant TCR convergence in the Th2A-like or Th2reg-like subsets, which could be due to the greater diversities of repertoires in these subsets that would require deeper sampling to detect any convergence.

Globally, we found that OIT induced a reduction of the frequency of CD154^+^ and CD137^+^ T cells and the expression of Th2 signatures in response to peanut antigen stimulation. In addition, we found that the TCR repertoires of peanut-reactive cells in peripheral blood were stable over time. These observations suggest that OIT acts predominantly via the suppression of functional phenotypes rather than by clonal deletion or TCR-biased sequestration away from the periphery. This result corroborates those of 2 previous studies that reported the emergence of anergic signatures in peanut-specific T cells over time in OIT and provides insight into previous reports of decreases in circulating Th2 cell frequencies following OIT (7, 17, 18, 21, 22). Nevertheless, future studies with comparatively deeper sequencing approaches and larger cohorts could further refine these observations of the influence of OIT on the peanut-reactive TCR repertoire in peripheral blood as well as among tissue-resident cell populations.

We also show how the 6 subsets of Th1, Th2, and Th17 cells identified in this study responded over the course of OIT. We observed OIT-induced suppression of Th2 and Th1 gene modules among Th2A-like and Th1-conv, but not Tfh-like, clonotypes. Strikingly, we found that the expression of select cytokines among Tfh2-like cells, but not Th2A-like cells, was correlated with peanut-specific IgE levels, suggesting that this subset may directly influence the peanut-specific IgE response. Last, we observed that the suppression of Th2 module expression in Th2A-like clonotypes was associated with clinical outcome. Our findings indicate that OIT modulates only a subset of peanut-reactive T cells and that the T cells most responsible for Ig class-switching and B cell help may be the least altered by treatment, highlighting the difficulty of achieving a sustained beneficial clinical outcome.

With respect to therapeutic outcomes, we found that an unsupervised composite score of all gene modules, derived using only data from cells isolated before treatment (baseline), corresponded strongly with treatment failure and was not modulated by OIT. This score was driven largely by markers of T cell activation such as *OX40*, *OX40L*, and *STAT1*, as well as by Th1 and Th17 genes (Supplemental Figure 14C). We surmised that high levels of baseline T cell activation could limit the effectiveness of OIT because of increased inflammation or altered gastrointestinal permeability (potentially triggered by Th17 responses; ref. 53). Assessments of genomic or immunologic features associated with clinical outcomes in OIT are scarce, but Th17 cells have been reported to play a role in atopic disease, with some preliminary evidence suggesting that these cells are modulated by OIT (25, 54–56). Similarly, OX40 and OX40L have also been implicated in atopic dermatitis and asthma and represent a possible therapeutic target (57, 58).

Moreover, while some of the top gene modules in the composite score were highly enriched among Th1 and Th17 subsets (e.g., the OX40L module), many were also expressed in other compartments of CD154^+^ or CD137^+^ cells (Supplemental Figure 16), including CD154^+^ cells not classified as any of the Th subtypes. This was the case for the GPR15 and STAT1 signaling modules (Supplemental Figure 16B). GPR15 has been highlighted as an esophagus-homing and colon-homing receptor in CD4^+^ T cells (42, 59), and STAT1 (along with GBP4 and GBP1, also included in the same module) is associated with response to IFN (60, 61). Taken together, these results suggest that altered gastrointestinal permeability and inflammatory responses in diverse populations of peanut-reactive T cells may influence the likelihood of a favorable response to OIT.

Although Tregs have been described by others as a correlate of a favorable clinical outcome in peanut OIT (19, 20), we did not find evidence for sustained peanut-reactive Treg induction during treatment. We found a lack of significant Treg induction both by clonotype frequency and by Treg gene expression levels, and we were able to assess this phenomenon within multiple Treg subtypes. Discrepancies between our results and those of prior studies could reflect differences in stimulation conditions and strategies for identifying antigen-specific Tregs (19). These differences should motivate further efforts toward elucidating the role of peanut-reactive Tregs in OIT.

Here, we analyzed peanut-reactive CD4^+^ T cells obtained from peripheral blood. A substantial fraction of peanut-reactive T cells is likely to establish residency in tissues, including the gastrointestinal tract and lymphoid organ tissues, but samples from these tissues cannot be as easily obtained. Thus, the impact of OIT on the phenotype and repertoire of tissue-resident peanut-reactive T cells remains unexplored in this study. Despite this limitation, the study of peripheral peanut-reactive T cells during OIT has considerable translational value, as changes in the peripheral blood can be easily monitored in a clinical setting. Moreover, we and others have successfully identified clinically relevant responses in peanut-reactive T cells from peripheral blood during OIT (17, 22, 62).

The methods we used in this study combined FACS-based enrichment of antigen-activated T cells with single-cell RNA-Seq and TCR sequencing as a framework for profiling antigen-reactive T cells without the use of tetramer reagents. By enriching peanut-reactive T cells on the basis of CD154 and CD137 expression, it is likely that our data included some fraction of nonspecifically activated T cells. By integrating data on TCR sequences, however, we identified T cell states that were associated with clonally expanded, peanut-reactive T cells, thereby minimizing the effects of nonspecifically activated T cells. We believe this framework could be used to identify likely antigen-reactive T cells in other disease contexts.

We believe this work has implications for the study of human T cell biology as well as mechanistic actions of OIT. First, the methodology implemented here provides a framework for the design and analysis of paired TCR and transcriptomic data of antigen-reactive T cells, and this substantial set of human single-cell data provides a useful reference for future studies. Using this framework, we detected significant heterogeneity within the peanut-reactive CD154^+^ T cell compartment and highlighted potential roles for TCR-epitope interactions in skewing the T cell phenotype. Second, our data reveal several features of OIT that merit further investigation. Based on our data, OIT did not appear to delete peanut-reactive Th2 clones; these findings point to selective clonal suppression, rather than deletion, as a major mechanism of OIT and highlight why sustained tolerance may be difficult to achieve. Furthermore, we found that failure to respond to OIT was reflected in a broad baseline activation signature, highly expressed in Th17 and other T cells, that was resistant to modulation by OIT. Future prospective OIT studies could evaluate this signature as a predictor of treatment success.

In summary, we used single-cell RNA-Seq and TCR clonotyping to reveal a complex set of highly distinct peanut-reactive Th cell phenotypes, beyond the effector Th2 phenotype, that are relevant to the efficacy of OIT. Future therapeutic modalities that either target these diverse phenotypes and inflammatory pathways, such as Tfh, Th17, OX40-OX40L, or that appreciably delete peanut-specific Th2A and Tfh2-like cells, may be more likely to promote sustained tolerance in food allergy than allergen-based approaches alone.

## Methods

### Patients.

Individuals with peanut allergy aged 7 years and older were enrolled in a peanut OIT trial (ClinicalTrials.gov identifier, NCT01750879) at the MGH Food Allergy Center. Study participants with a previous diagnosis of peanut allergy, a history of peanut-induced reactions consistent with immediate hypersensitivity, and confirmatory peanut- and Ara h 2–specific plasma IgE concentrations (peanut-specific IgE >5 kU/L, Ara h 2–specific IgE >0.35 kU/L; ImmunoCAP; Thermo Fisher Scientific) underwent a double-blind, placebo-controlled food challenge (DBPCFC). Increasing peanut protein doses were administered every 20 minutes to a maximum dose of 300 mg according to the following doses: 3, 10, 30, 100, and 300 mg, for a cumulative total of 443 mg. Patients who had an objective allergic reaction during the challenge were eligible for inclusion in the study. Demographic classifications were self-reported by the study participants.

### OIT study.

The main objective of this phase I/II, double-blind, placebo-controlled interventional study was to provide safety and mechanistic data on OIT for individuals with IgE-mediated peanut allergy. Enrolled patients were randomized to receive either treatment (peanut flour) or placebo (roasted oat flour) at a ratio of 3:1. The treatment consisted of a modified rush protocol, followed by a buildup phase that lasted 44 weeks or until the patient could tolerate a 4000 mg dose of peanut protein, whichever came first. The treatment dose was administered daily, and dosing escalation was incremental, based on previous OIT studies (20, 26), and was done every 2 weeks. After the buildup phase, the patients entered a maintenance phase, in which treatment was continued for 12 weeks at the highest tolerated dose for each patient. Finally, the patients underwent an avoidance phase consisting of 12 weeks off therapy with strict avoidance of dietary peanut protein in order to assess the durability of any desensitization resulting from OIT. During each phase of the study, a blood sample was taken (4 samples per patient) 2 weeks prior to the start of treatment at baseline, 14 weeks into the buildup phase, 8 weeks into the maintenance phase, and 8 weeks into the avoidance phase.

Clinical assessments were made by DBPCFC at baseline (DBPCFC1), at the end of 12 weeks of maintenance therapy (DBPCFC2), and at the end of 12 weeks of avoidance (DBPCFC3) (20). Clinical outcomes were defined as follows: treatment failure (failure to achieve the minimum maintenance dose of 600 mg peanut protein by 12 months, or an eliciting dose of less than 1443 mg at DBPCFC2, or less than 443 mg at DBPCFC3, or less than 10-fold higher than at DBPCFC1); partial tolerance (eliciting dose of less than 4430 mg at DBPCFC3 but of at least 443 mg and more than 10-fold higher than at DBPCFC1); and tolerance (ingestion of 4430 mg of peanut protein at DBPCFC3 without symptoms).

### Cell purification and sorting.

After a blood sample was collected, PBMCs were isolated by density-gradient centrifugation (Ficoll-Paque Plus; GE Healthcare) and cryopreserved in FBS with 10% DMSO. After the study was completed, for each of the 12 patients, PBMCs from all 4 time points (15 × 10^6^ to 30 × 10^6^ PBMCs per time point) were simultaneously thawed, washed with PBS, and cultured in AIM-V Medium (Gibco, Thermo Fisher Scientific) with 100 μg/mL peanut protein extract for 20 hours, at a density of 5 × 10^6^ PBMCs in 1 mL medium per well in 24-well plates. Peanut protein extract was prepared by agitation of defatted peanut flour (Golden Peanut and Tree Nuts) with PBS, centrifugation, and sterile filtration. The endotoxin concentration in the peanut protein extract was assessed to be 6 EU/mg, using a LAL Endotoxin Quantitation Kit (Thermo Fisher Scientific; catalog 88282). This 6 EU/mg concentration was lower than that found in commercially available endotoxin-depleted preparations of the purified peanut proteins Ara h 1 and Ara h 2 (Indoor Biotechnologies; LTN-AH1-1 and LTN-AH2-1). Furthermore, the endotoxin concentration in the PBMC cultures with peanut protein extract was 0.6 EU/mL, which is comparable to the endotoxin limit for eluates from medical devices (0.5 EU/mL) as determined by the FDA (63). Anti–CD154-PE antibody (BD Biosciences; clone TRAP1) was added to the cultures at a 1:50 dilution (20 μL/well) for the last 3 hours. After harvesting, the cells were labeled with anti–CD3-AF700 (BD Biosciences; UCHT1); anti–CD4-APC-Cy7 (BD Biosciences; RPA-T4); anti–CD45RA-PE-Cy7 (BD Biosciences; HI100); anti–CD154-PE (BD Biosciences; TRAP1); anti–CD137-APC (BD Biosciences; clone 4B4-1); and Live/Dead Fixable Blue Stain (Thermo Fisher Scientific; catalog L23105). Cells were then sorted with a FACSAria Fusion instrument (BD Biosciences). Cells were gated as live singlet CD3^+^CD4^+^CD45RA^–^ cells and then sorted as either CD154^+^CD137^+/–^ (referred to hereafter as CD154^+^), CD154-CD137^+^ (referred to hereafter as CD137^+^), or CD154^–^CD137^–^.

### Single-cell RNA-Seq.

Sorted subsets of CD4^+^ memory T cells were processed for single-cell RNA-Seq using the Seq-Well platform as previously described (36). A portion of each cDNA library was reserved for paired TCRα/β enrichment. The rest was barcoded and amplified using the Nextera XT kit and sequenced on the Illumina NovaSeq.

Raw read processing was performed as described in Macosko et al. (64). Briefly, sequencing reads were aligned to the hg38 reference human genome, collapsed by unique molecular identifier (UMI), and counted to obtain a digital gene expression matrix of cells versus genes. These counts were then filtered to exclude any cells with fewer than 1000 genes or 2000 UMIs and normalized by library size per cell and log transformation. (For the rare Th subset analysis, which required more cells, a filter of 500 genes and 1000 UMIs was used.) After this filtering step, a total of 74,646 CD154^+^ cells were recovered from 12 patients, 41,186 CD137^+^ cells from 11 patients, and 18,297 CD154^–^CD137^–^ cells from 6 patients.

### Paired single-cell TCRαβ sequencing.

Paired TCR sequencing was performed according to Tu et al. (37). Briefly, following cDNA amplification, biotinylated capture probes for human *TRAC* and *TRBC* transcript regions were annealed to cDNA. Magnetic streptavidin beads were used to enrich the bound TCR sequences, which were then further amplified using human V-region primers and prepared for sequencing using Nextera sequencing handles. Libraries were sequenced on an Illumina MiSeq using 150 bp length reads.

TCR sequencing reads were preprocessed according to the method in Tu et al. (37). In short, reads were mapped to TCRV and TCRJ IMGT reference sequences via IgBlast, and V and J calls with “strong plurality” (wherein the ratios of the most frequent V and J calls to the second most frequent calls were at least 0.6) were retained. CDR3 sequences were called by identifying the 104-cysteine and the 118-phenylalanine according to IMGT references and translating the amino acid sequences in between those residues. Processed TCR sequences were then paired with the single-cell transcriptomic data via the cell barcodes.

### Visualization of single-cell RNA-Seq data.

Visualization of single-cell transcriptomes was done with UMAP (65) with the Python package scanpy. Prior to visualization, the normalized gene expression data were transformed using a standard “regress-out” approach to mitigate batch effects, whereby a multiple linear regression was performed on all genes with 2 covariates that could be batch associated (number of transcripts per cell, and percentage of transcripts aligning to the mitochondrial chromosome). The residuals from this regression were taken as the transformed data. Next, PCA was performed, and the top 10 components were used to generate a UMAP visualization.

### Gene module discovery.

Coexpressed gene modules were generated using the sparse PCA approach described by Witten et al. and implemented in the R package PMA (38). This unsupervised method utilizes an L1 norm penalty on loadings in each component to introduce sparsity. Prior to running the sparse PCA, the gene expression matrix was randomly downsampled to have an equal number of cells from all samples to prevent the results from being skewed by a subset of the samples. Genes were filtered down to the union of immune genes (defined by the set of gene lists available on ImmPort at https://www.immport.org/shared/genelists) and the variable genes in the data set (defined using the R package Seurat). Finally, the gene expression data were scaled with respect to genes, and sparse PCA was run using the command “SPC.” Gene module scores were calculated as the scaled gene expression input matrix multiplied by the outputted loadings matrix “v.” The first 50 gene modules were retained for downstream analysis.

Cells were classified as “expressing” or “not expressing” a module using a simple thresholding strategy. The distribution of module scores of CD154–CD137^–^ cells was used as a negative control, and a threshold was set at the point where 0.2% of CD154^–^CD137^–^ cells were in the positive population. Cells with a module score above the threshold were labeled as “expressing” that module. Modules in which at least 60% of the expressing cells were from a single patient were removed from the downstream analysis. For analysis with the Treg module (module 1), which was more highly expressed among CD154^–^CD137^–^ cells than the other Th modules, we instead identified a threshold score (module score = 2.0) that most accurately separated the positive and negative cell populations.

### Identification of Th subtypes.

All CD154^+^ and CD137^+^ cell transcriptomes were classified as Th1, Th2, or Th17 using the criteria for module expression detailed above (see *Gene module discovery*) for the Th1, Th2, and Th17 gene modules. If a cell expressed more than 1 Th module, it was assigned to the module with the highest *z* score (compared with the distribution of all CD154^+^ and CD137^+^ cells). Then, each individual Th class (Th1, Th2, and Th17 cells) was separately visualized by UMAP and clustered by Louvain clustering using the R package Seurat. For Treg analysis, all CD154^+^ and CD137^+^ cells, including those with Th1, Th2, or Th17 signatures, were considered using the criteria for module expression in Tregs (see *Gene module discovery*).

### Distance analysis of TCR sequences.

Pairwise distance of TCRβ CDR3 sequences was evaluated using the TCRdist method published by Dash et al. (47). Briefly, for 2 TCRβ CDR3 amino acid sequences of the same length, each residue position was compared, and a penalty was assessed for every mismatch. The penalty for 2 different amino acid residues i and j was assessed using the BLOSUM62 matrix and was defined as min(4 – BLOSUM62[i, j], 4). Each substitution thus incurred a penalty between 1 and 4. The overall distance between 2 CDR3s was calculated as the sum of penalties at all positions. In the case of 2 CDR3s of unequal length, the sequences were aligned in all possible ways and the minimum overall penalty was taken, with each gap incurring a penalty of 8.

### Likelihood-based association between TCR and Th subtype.

Likelihood-based analysis was used to determine the tightness of association between Th subset and the TCRβ CDR3 sequence. A log-likelihood ratio was defined as log_2_(*P/P0*), where *P* is the probability of 2 cells belonging to the same Th subset if they were drawn randomly from all cells sharing the same TCRβ CDR3 sequence (without replacement), and *P0* is the probability of 2 cells belonging to the same Th subset if they were drawn randomly from all cells. *P0* represents the prior probability without the constraint of TCR information; thus, the ratio *P/P0* represents the gain in likelihood due to the knowledge of the TCR sequence. This analysis was constrained to consider all pairs of cells from the same patient.

### Analysis of gene module suppression in Th and Treg clones.

To quantify the suppression or induction of relevant Th and Treg gene and gene module expression over time, we first identified Th (Th1, Th2, or Th17) and Treg clones, defined as clonotypes in which the relevant module (e.g., Th2) was expressed in at least 1 cell at any time point. This approach allowed us to expand our analysis to peanut-reactive cells that may gain or lose Th or Treg phenotypes over the course of OIT. Mean expression of genes or gene modules was then calculated for each patient at each time point. For analysis of Treg clones, patient 105 (full tolerance) was omitted, since data from CD137^+^ T cells were not available (see *Single-cell RNA-Seq*).

### Longitudinal analysis of individual clonotypes.

Temporal analysis of individual clonotypes over the course of OIT involved 2 analyses: (a) determination of the distribution of time points at which each clonotype was detected, and (b) assessment of module expression of clonotypes within Th subtypes. For the former, CD154^+^ or CD137^+^ clones were filtered to identify those with at least 4 cells in that sorted subset. Then, the time points covered by the cells were tabulated, and the clonotype was classified as having a specific temporal pattern (e.g., baseline, buildup, avoidance). For the latter, clonotypes were filtered to identify those with at least 2 cells in the combined CD154^+^ and CD137^+^ compartments at each time point. Each clonotype was then assigned to 1 of the 6 Th subtypes (or no subtype) on the basis of the most frequent Th subtype that its cells mapped to (see *Identification of Th subtypes*). At each time point, the fraction of cells within each clonotype expressing the relevant module (Th1, Th2, or Th17) was counted, relative to the total number of cells of that clonotype at that time point. Fractional expression was used instead of module scores to normalize for clonotype- or patient-driven differences in the dynamic range of module expression.

### Baseline signature of all modules using PCA.

PCA was used to identify broad immune signatures associated with clinical outcome. Mean module scores of the 50 gene modules (minus the 7 modules associated with a single patient; see *Gene module discovery*) were computed for each patient at each time point. Averages at baseline were used to compute the principal components. The first principal component (PC1), or the component explaining the largest amount of variance, was then applied to module averages of other time points.

### Spearman’s correlation of treatment outcome and module expression.

To investigate whether treatment outcome was correlated with expression of modules enriched in PC1, we assigned numerical values to each of the outcomes (tolerance as 2, partial tolerance as 1, and treatment failure as 0) to represent an ordinal relationship between the outcomes. Spearman’s correlation between mean module expression (by patient and cell subset) and outcomes was calculated. The corresponding unadjusted *P* values are reported.

### Data availability.

FASTQ file format data related to human samples are available through the NCBI’s dbGaP (accession no. phs001897.v2.p1). Processed gene expression and associated TCR clonotype data are available through the NCBI’s Gene Expression Omnibus (GEO) database (GEO GSE158667). Processed data files and associated metadata tables for Figures 1–6 are available on https://github.com/mitlovelab/, under GEO GSE158667.

### Code availability.

R, python, and MATLAB scripts for processing TCR sequencing data and generating all analyses, as well as all updates, are available on https://github.com/mitlovelab/PNOIT2_scRNAseq and (commit ID: 90ecf6f8a35f7d0fcace61b7b488da87dd9a6562).

### Statistics.

Paired or unpaired Wilcoxon rank sum tests were performed for comparisons between 2 groups. Spearman’s correlation was assessed for association of clinical group rankings with phenotypes (e.g., module scores) and for association of *IL4* and *IL5* expression with peanut-specific IgE titer. A 2-sided, χ^2^ proportion test with 1 degree of freedom was used for the TCR distance analysis. All statistical tests were performed as 2-sided tests unless otherwise specified. Box plots were plotted with the standard visualization of 25th and 75th percentiles for the lower and upper hinges and at most 1.5 times the IQR for the whisker lengths. *P* values of less than 0.05 were considered statistically significant. All adjusted *P* values were calculated using Bonferroni’s correction unless stated otherwise.

### Study approval.

All participants were recruited with informed consent, and the study was approved by the IRB of Mass General Brigham Healthcare (protocol 2012P002153).

## Author contributions

WGS and JCL conceptualized the study. WGS conducted the clinical trial (NCT01750879). BM, AAT, BR, and PMP conducted experiments. BM, AAT, DMM, and NPS analyzed the data. TMG and JHG conducted experiments to develop and validate the methods. BM, AAT, BR, DMM, WGS, and JCL wrote and edited the manuscript. BM and AAT contributed equally to this research as co–first authors. The order of appearance of the co–first authors was based on the timeline of their contributions to the work.

## Supplementary Material

Supplemental data

Supplemental Tables 3 and 4

## Version 1 11/23/2021

In-Press Preview

## Version 2 01/18/2022

Electronic publication
