# Supplementary material for: Peanut oral immunotherapy differentially suppresses clonally distinct subsets of T helper cells
Source: J Clin Invest. 2022 Jan 18;132(2):e150634. doi: 10.1172/JCI150634 (PMC8759778; doi:10.1172/JCI150634)
Supplement: Supplemental data [file jci-132-150634-s170.pdf]

Supplementary Table 1. Patient demographics and baseline characteristics.

Patient ID	Treatment Group	Age	Gender	Race	Peanut IgE (kU/L)	Peanut IgG4 (mg/L)	Total IgE (kU/L)	Skin prick test, wheal (mm)
105	Treatment	22	Female	White	44.4	0.49	109	7.5
106	Treatment	32	Female	White	4.5	0.29	40.8	10
111	Treatment	22	Female	White	20.9	0.16	169	5
33	Treatment	16	Female	White	84.1	0.54	216	10
90	Treatment	9	Male	White	159	0.85	338	14.5
93	Treatment	11	Male	White	40.9	1.86	208	7
69	Treatment	15	Male	White	11.2	0.16	141	13
95	Treatment	8	Male	White	451	1.71	1524	24
97	Treatment	36	Female	Asian	2.6	0.62	339	13.5
84	Placebo	22	Male	White	61.4	0.09	174	11
96	Placebo	10	Female	White	39.1	0.18	151	10
107	Placebo	22	Female	White	27.3	0.37	88.1	22

Supplementary Table 2. Patient clinical outcomes.

Patient ID	Treatment Group	Cumulative dose consumed at DBFC2	DBFC2 outcome	Cumulative dose consumed at DBFC3	DBFC3 outcome	Adverse event count	Therapeutic outcome
105	Treatment	4443	Pass	4440	Pass	605	Tolerance
106	Treatment	4443	Pass	4440	Pass	269	Tolerance
111	Treatment	4443	Pass	4440	Pass	61	Tolerance
33	Treatment	4443	Pass	4440	Fail	306	Partial tolerance
90	Treatment	4443	Pass	4440	Fail	60	Partial tolerance
93	Treatment	4443	Pass	4440	Fail	177	Partial tolerance
69	Treatment	943	Fail	40	Fail	101	Treatment failure
95	Treatment	4443	Fail	440	Fail	26	Treatment failure
97	Treatment	289.6	Fail	1440	Fail	497	Treatment failure
84	Placebo	443	Fail	--	--	81	Treatment failure
96	Placebo	143	Fail	--	--	42	Treatment failure
107	Placebo	943	Fail	--	--	25	Treatment failure

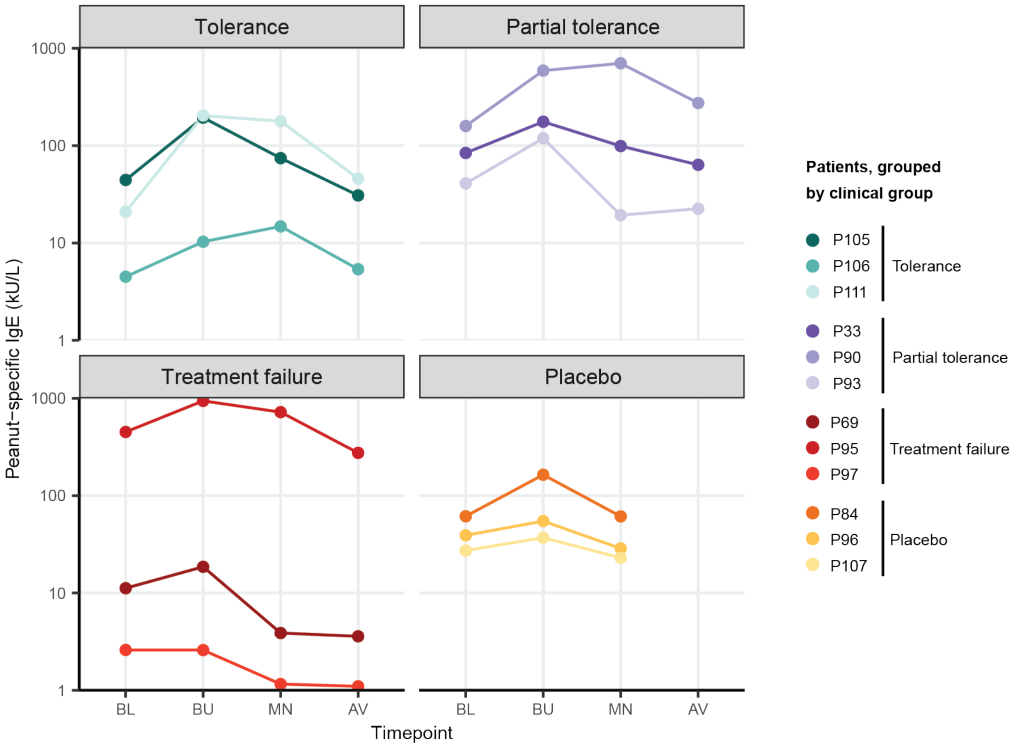

Supplementary Figure 1. Peanut-specific levels in plasma during OIT. Peanut-specific IgE measurements for each patient at each timepoint during OIT.

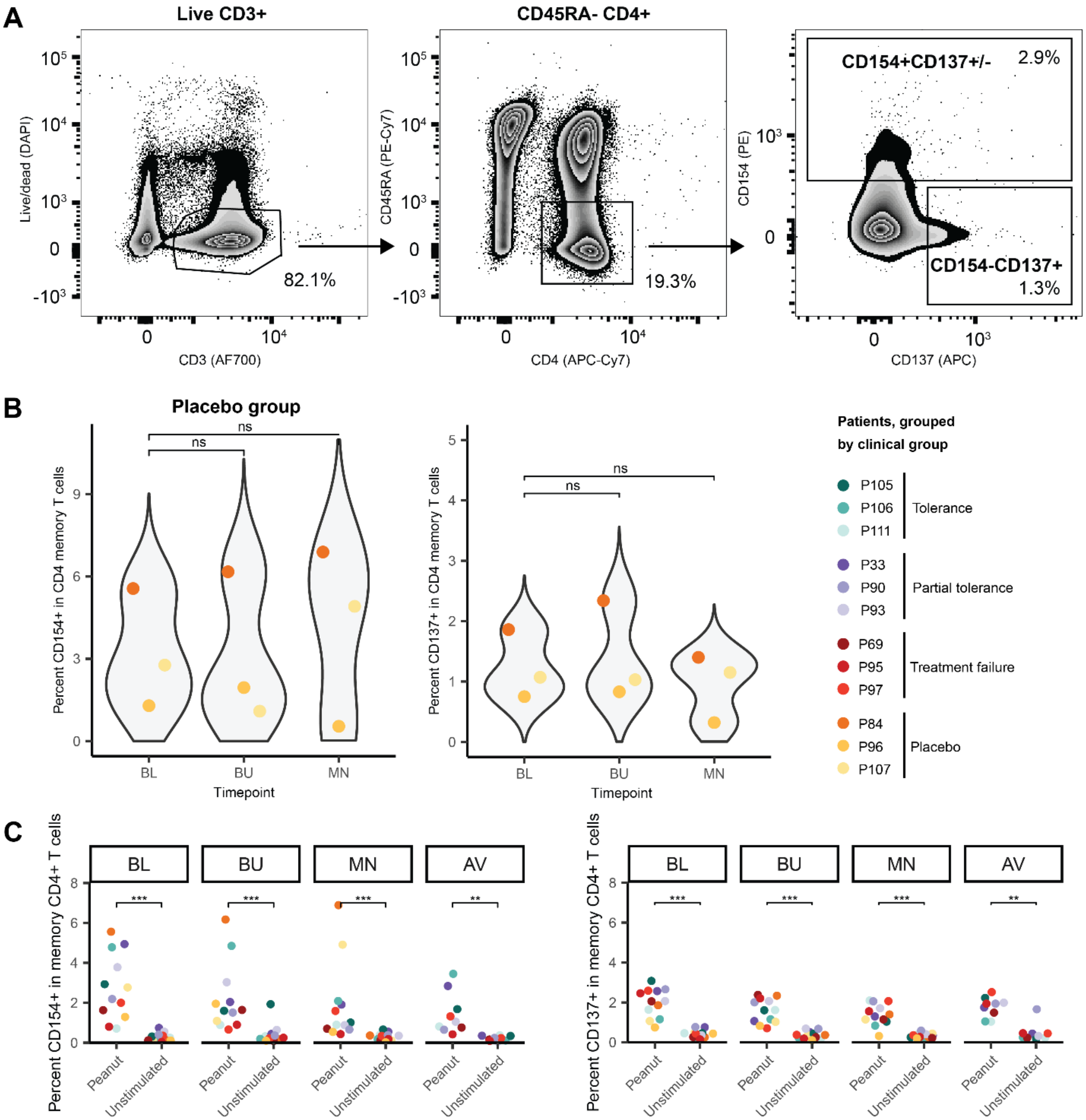

Supplementary Figure 2. Frequency of CD154+ and CD137+ peanut-reactive T cells. A, Gating strategy for CD154 and CD137 expression in CD4+ memory T cells from PBMC cultures stimulated with peanut antigen. Data are from a representative patient at baseline (n = 12 patients total). **B**, Percent of CD4+ memory T cells at each time point that are CD154+ (left) or CD137+ (right) in peanut-stimulated PBMC cultures from patients in the placebo group. **C,** Percent of CD154+ (left) or CD137+ (right) cells in CD4+ memory T cells in cultures stimulated with peanut antigen or without stimulation. P-values were calculated using a paired two-sided Wilcoxon rank-sum test and were adjusted with Bonferroni correction. '*' refers to an adjusted p-value of <0.05, "**" refers to adjusted p-value of <0.005, and "****" refers to adjusted p-value of <0.0005 (**B, C**).

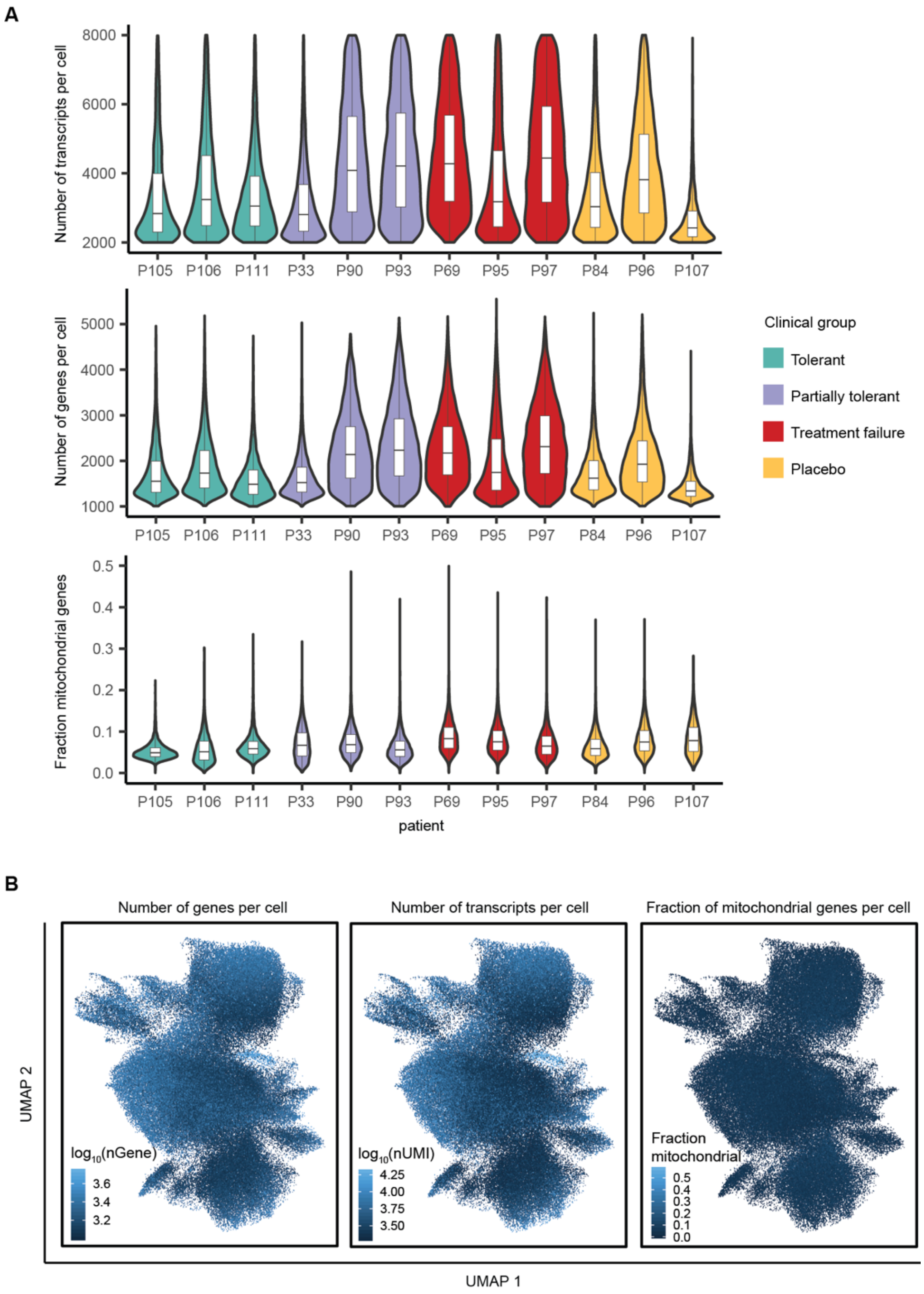

Supplementary Figure 3. Quality of single-cell RNA-Seq libraries. A, Quality control metrics of single-cell RNA sequencing libraries: number of unique molecular identifiers (UMIs) per cell (top), number of genes detected per cell (middle), and fraction of genes detected that were mitochondrial for each cell (bottom). Each violin represents all cells recovered from one patient. **B**, Quality control metrics overlaid on UMAP representation of the data as shown in **Figure 1**.

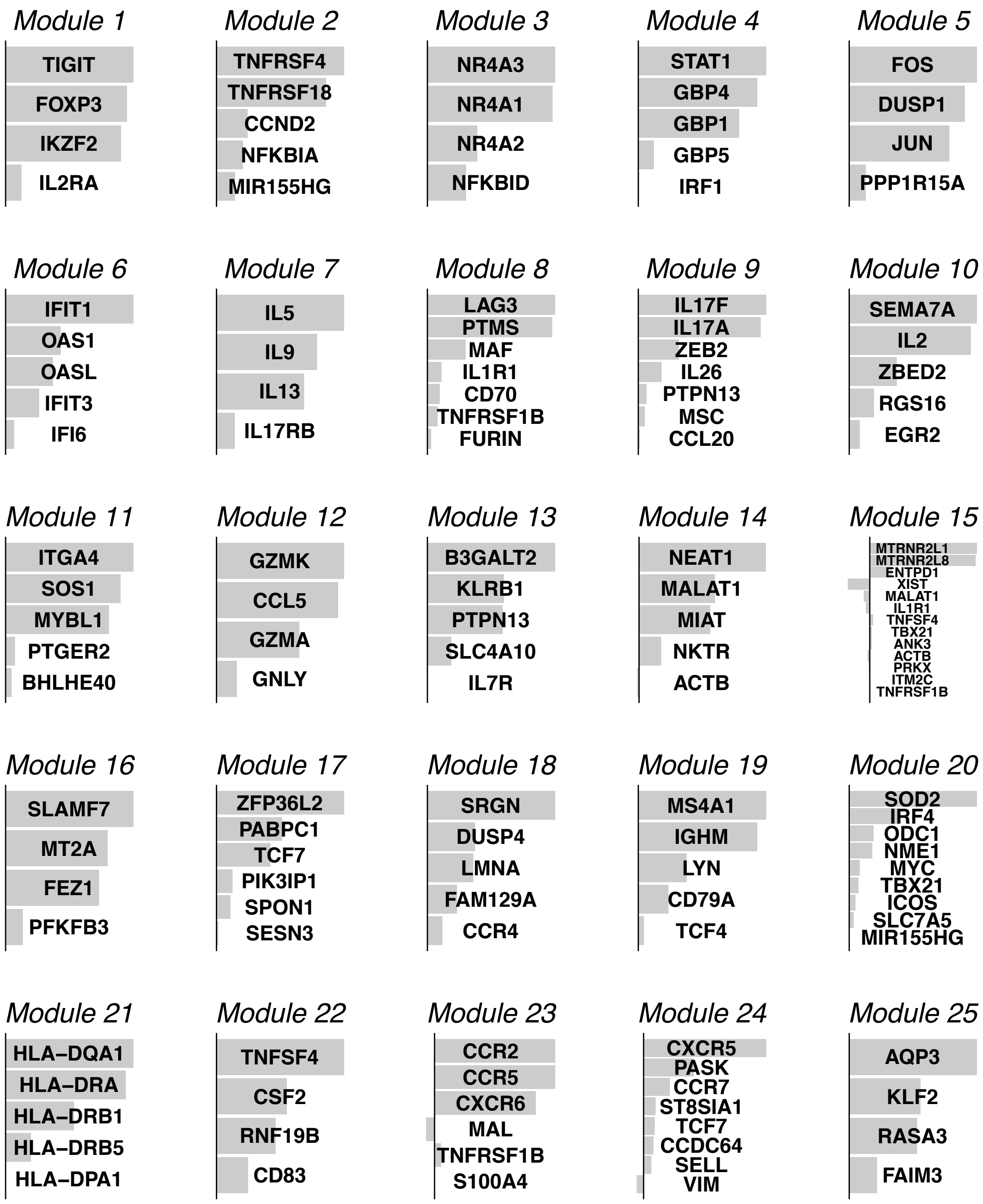

Supplementary Figure 4. Gene modules 1 through 25. Gene modules were discovered from immune-related and variable genes in an unsupervised manner using sparse PCA. For each module, the magnitude and direction of each bar represent the weight and sign of each gene in that module, respectively.

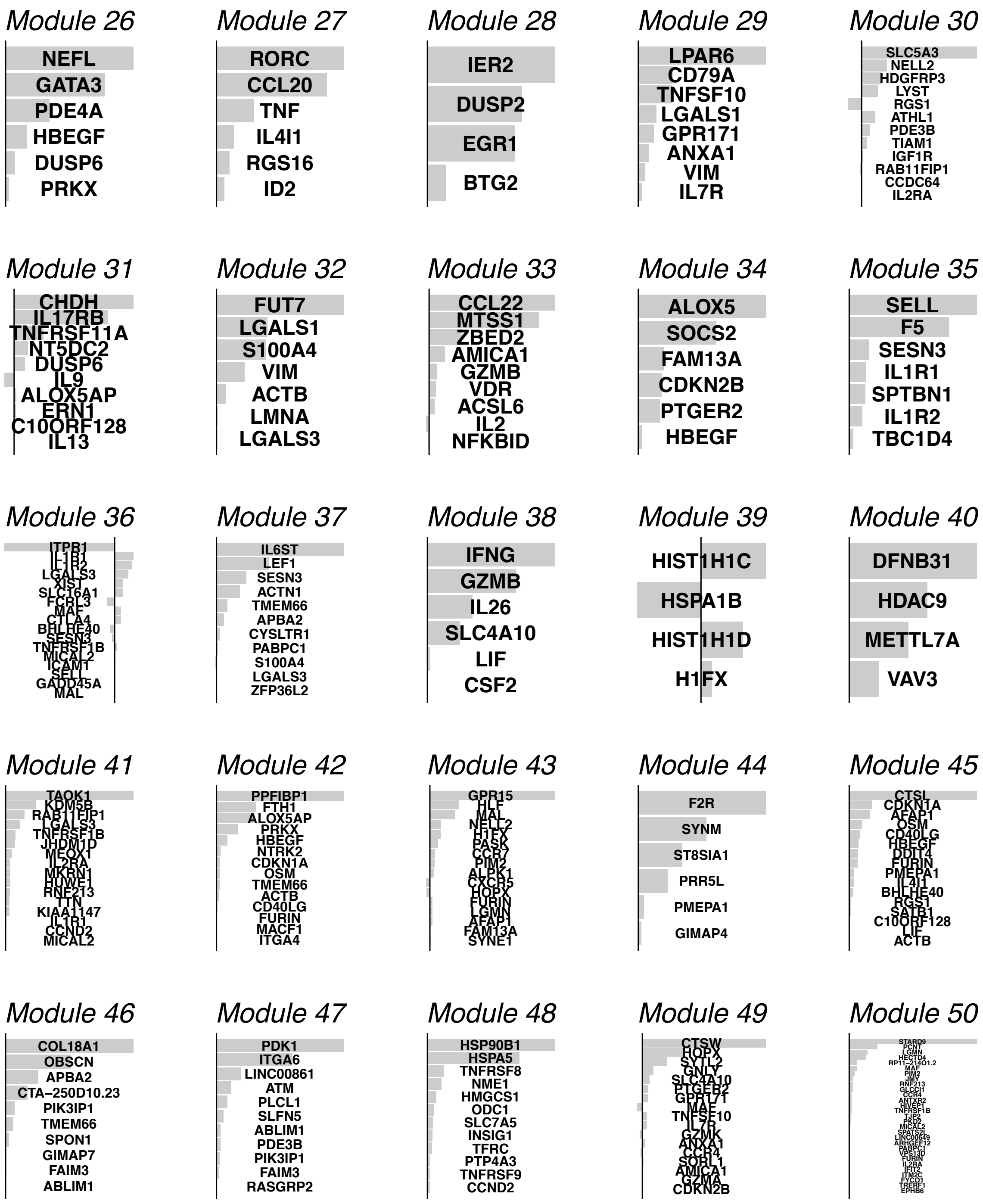

Supplementary Figure 5. Gene modules 26 through 50. Gene modules were discovered from immune-related and variable genes in an unsupervised manner using sparse PCA. For each module, the magnitude and direction of each bar represent the weight and sign of each gene in that module, respectively.

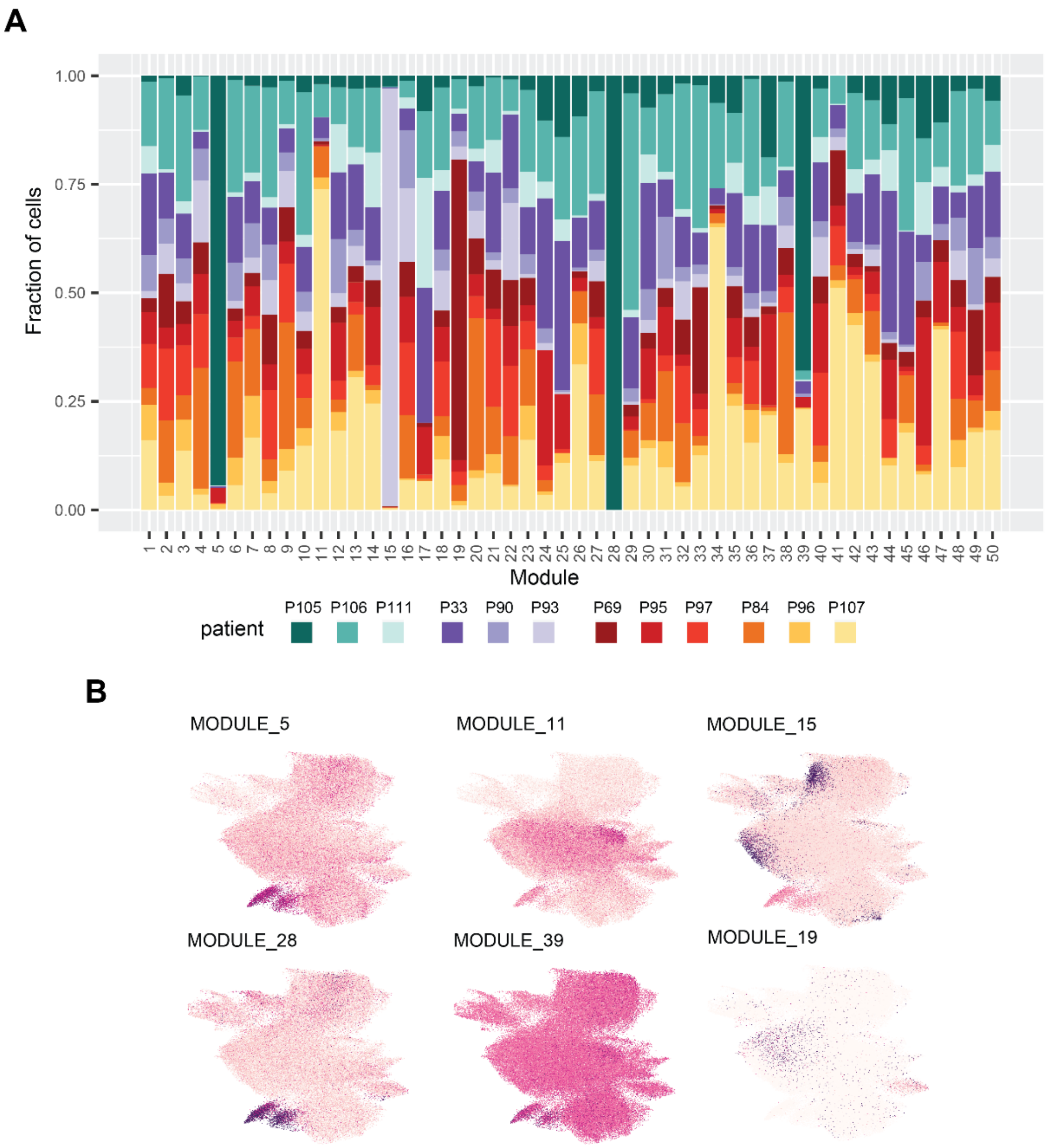

Supplementary Figure 6. Gene module expression across patients. A, Distribution of module-expressing cells by patient, for the 50 gene modules. “Module-expressing” cells were determined using the CD154-CD137- cells as a control, as described in **Methods**. **B**, UMAP overlay of module expression, and module loadings, for key patient-associated modules.

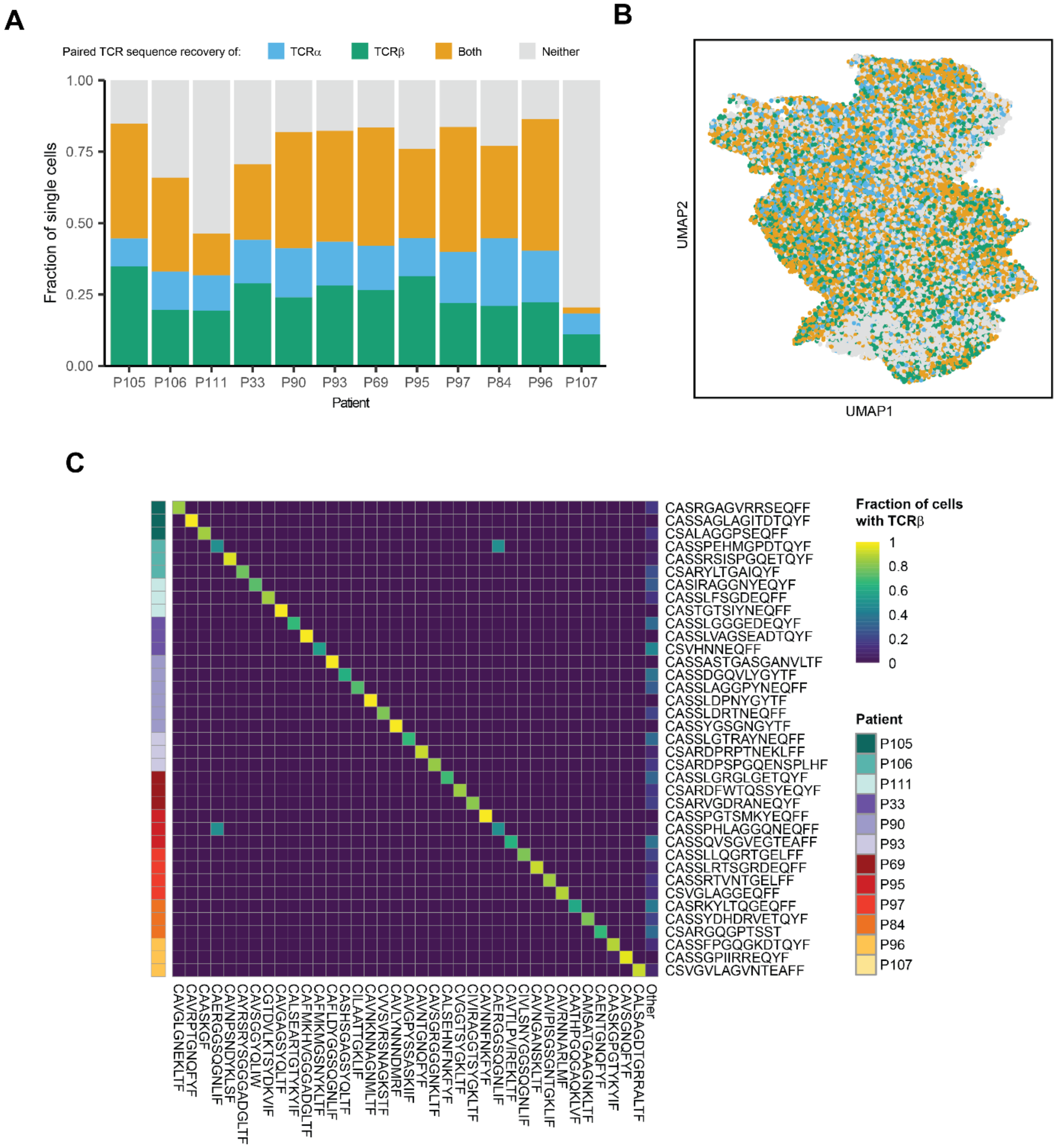

Supplementary Figure 7. Quality of single-cell TCRα/β sequences. A, Fraction of cells with each status of TCR sequence recovery for all cells obtained from each patient. **B**, UMAP overlay of TCR recovery status of every cell. **C**, TCRα pairing for the 30 most frequently detected TCRβ sequences. Within cells of each TCRβ sequence, the most frequent pairing TCRα sequences were plotted. The 3 most expanded TCRβ sequences from each patient are shown. The column "Other" captures the fraction of TCRα sequences that were not detected in at least 50% of cells of that TCRβ clonotype. Rows are annotated with the patient in which the TCRβ clonotype was most frequently detected.

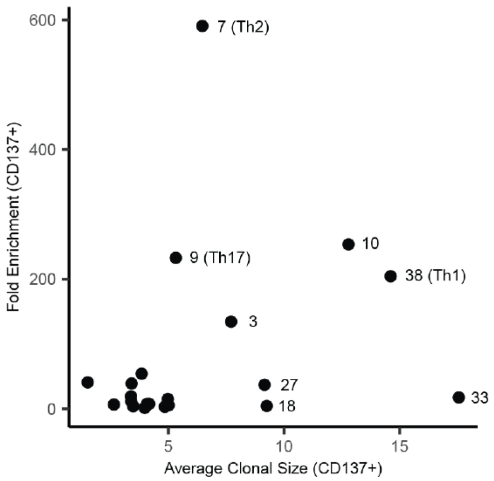

Supplementary Figure 8. **Mean clonal size and fold-change in mean module scores in CD137+ cells expressing each gene module**. Fold change is relative to module-expressing CD154-CD137- cells. Cells were classified as ‘expressing’ each module or not, relative to background expression (**Methods**). Clonal size was calculated with respect to all cells in the dataset. Data represent combined data from all patients at all timepoints.

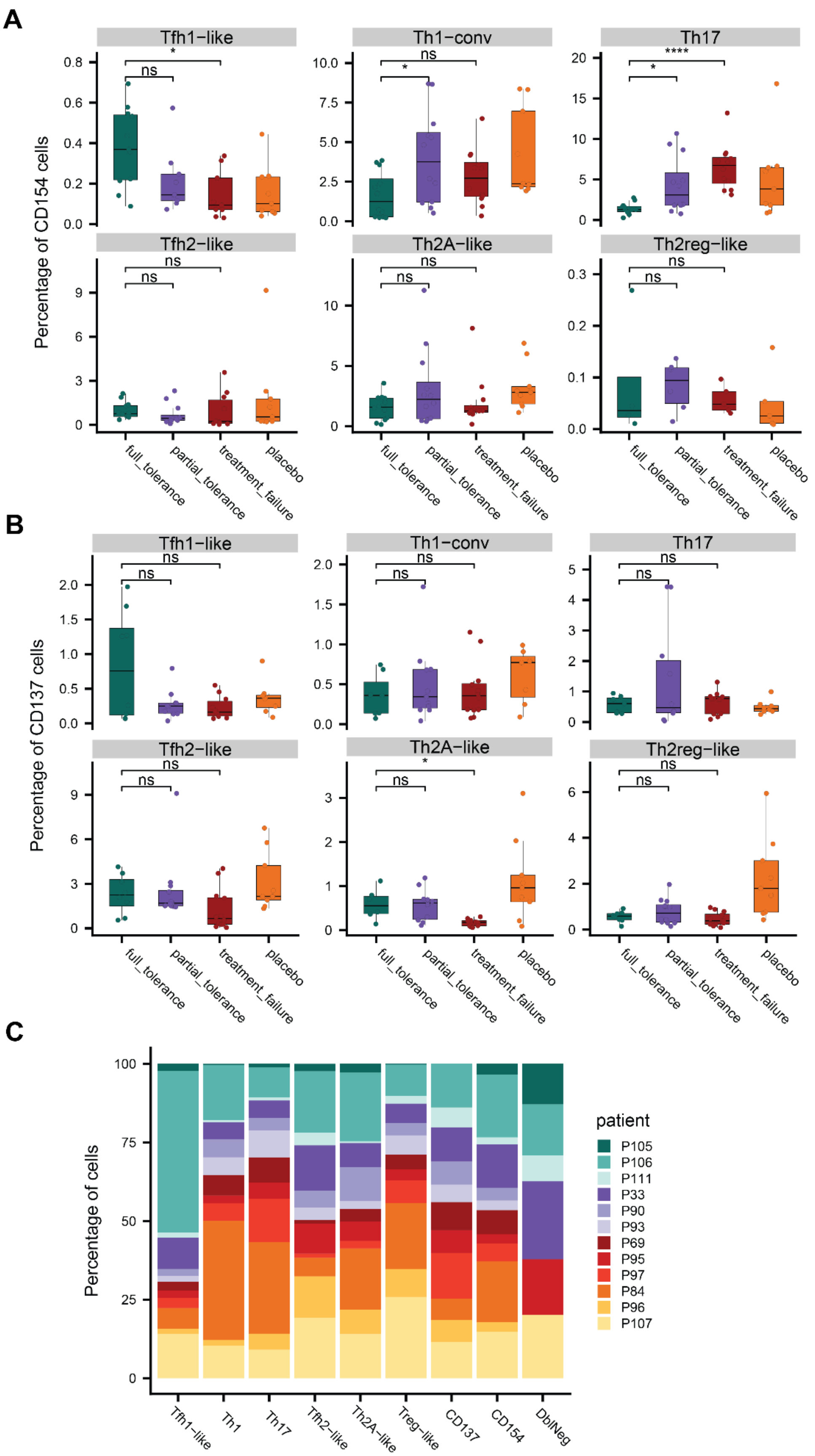

Supplementary Figure 9. Frequency of T helper subsets by clinical group. A, Frequency of cells of each T helper subtype among all CD154+ cells for each patient at each timepoint, grouped by clinical group. **B**, Frequency of cells of each T helper subtype among all CD137+ cells for each patient at each timepoint, grouped by clinical group. **C**, Fraction of cells comprising each T helper subtype (and all CD154+, CD137+, or CD154-

CD137- cells, for comparison) from each patient. P-values were calculated using a paired two-sided Wilcoxon rank-sum test and were adjusted with Bonferroni correction. “*” refers to an adjusted p-value of <0.05, “**” refers to adjusted p-value of <0.005, and “****” refers to adjusted p-value of <0.0005 (**A**-**B**).

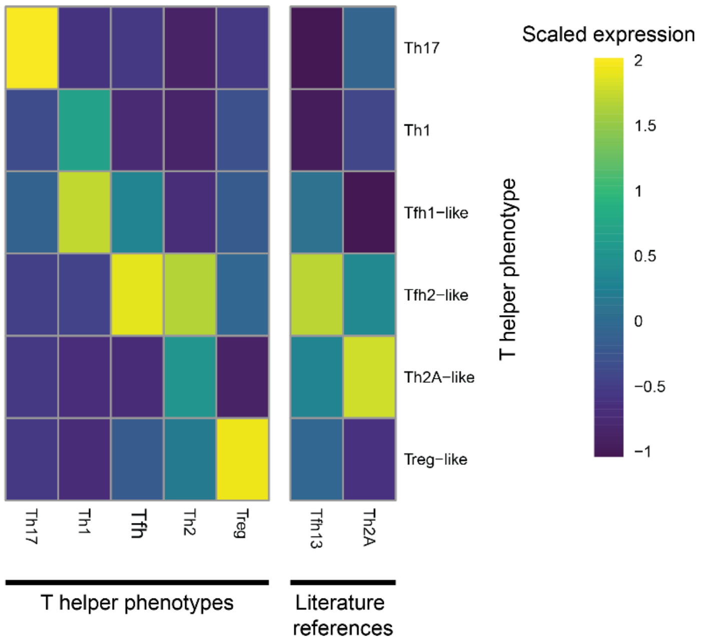

Supplementary Figure 10. Th1, Th2 and Th17 subsets express previously identified signatures. Mean expression scores of Th subset signatures identified in previous studies (**Supplementary Table** 4). Th subsets identified in **Figure** 4 (y-axis) were scored for each of the known or previously identified signatures (x-axis) using the AddModuleScore function in Seurat, then averaged within each subset.

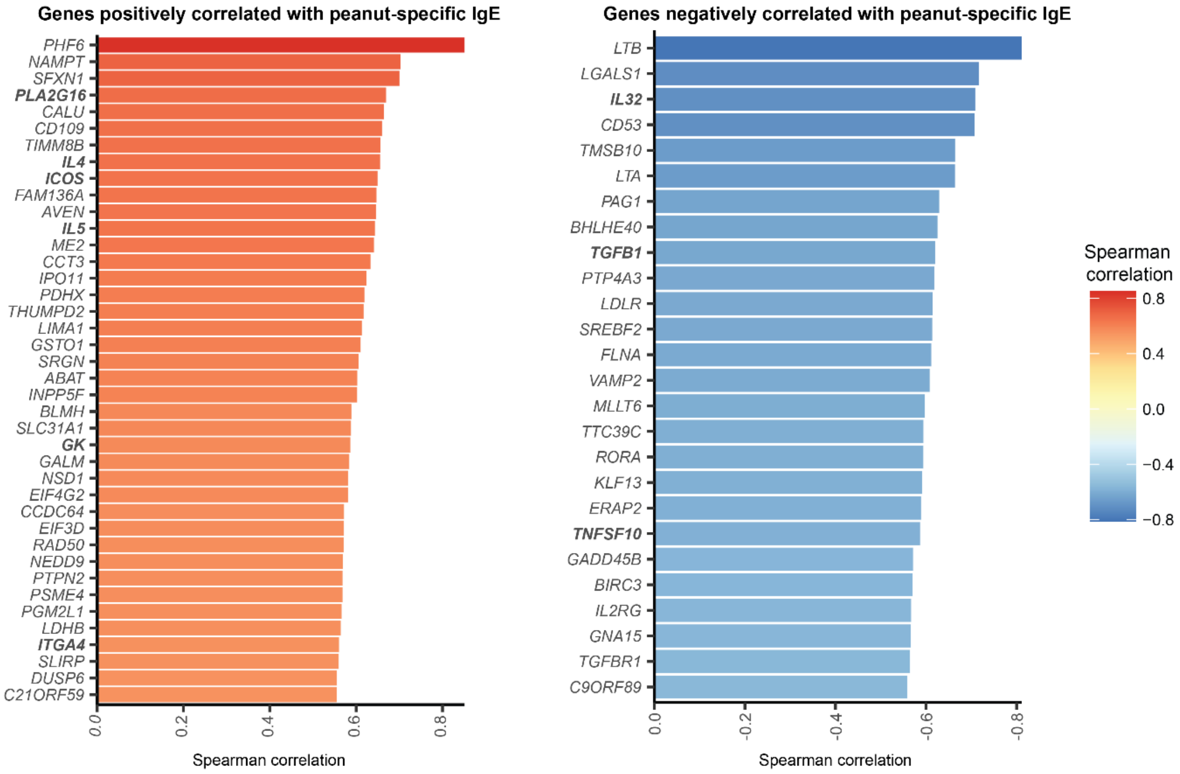

Supplementary Figure 11. Gene expression in Tfh2-like cells is correlated with peanut-specific IgE. Genes expressed by Tfh2-like cells that are significantly positively correlated or negatively correlated with peanut-specific IgE titers (adjusted p-value < 0.05). For each gene, the average expression by Tfh2-like cells present for each patient and timepoint was computed. The Spearman correlation (n = 34) between average gene expression and peanut-specific IgE titers was determined, and the associated p-values were adjusted using Benjamini-Hochberg correction.

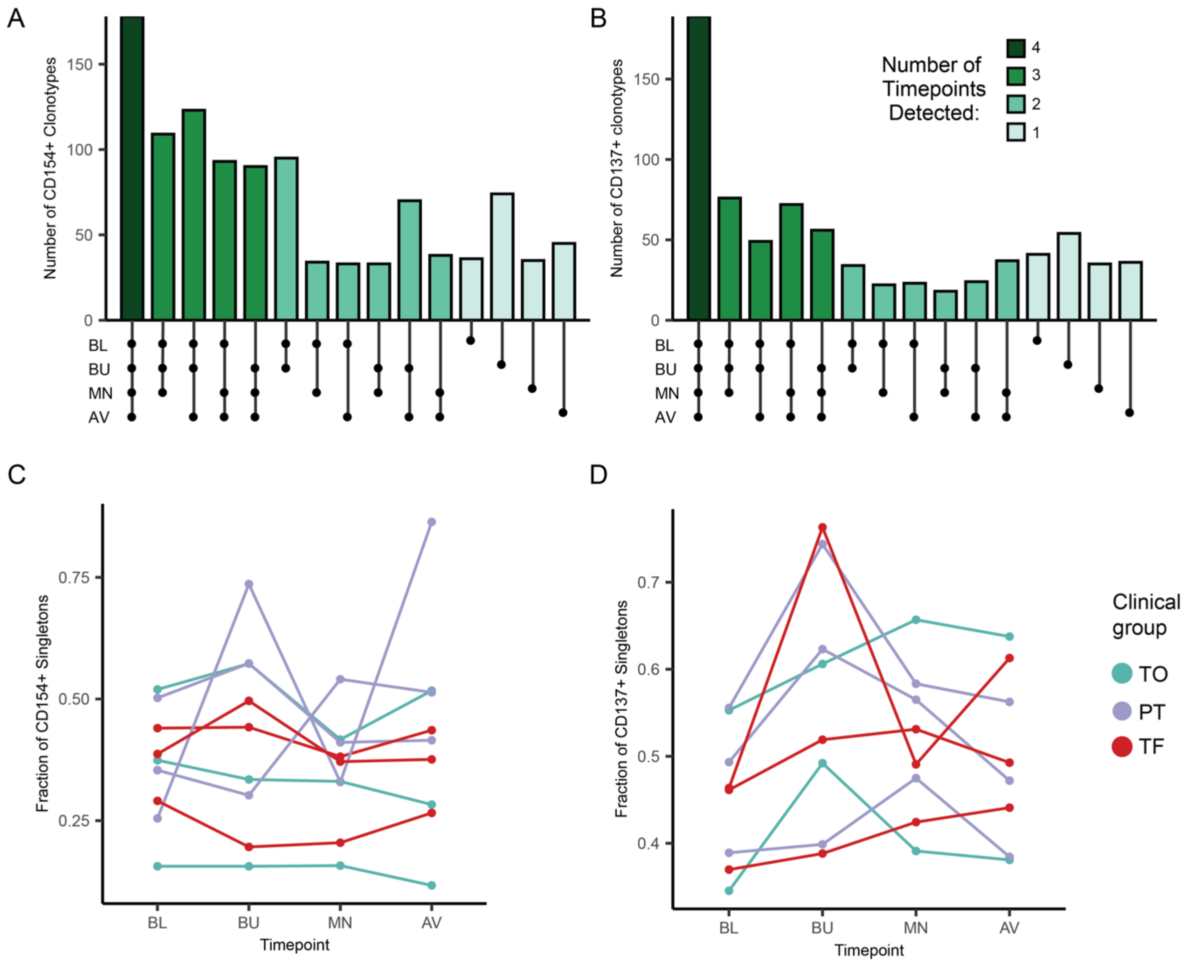

Supplementary Figure 12. Distributions of TCRβ clonotypes by timepoint. A-B, Number of TCRβ clonotypes detected at every possible combination of timepoints in (**A**) CD154+ and (**B**) CD137+ cells. Clones from all treatment-group (placebo group excluded) patients with at least 4 cells were included. **C-D**, Fraction of singletons (clonotypes with clonal size of one) detected within each patient and at each timepoint in (**C**) CD154+ and (**D**) CD137+ cells.

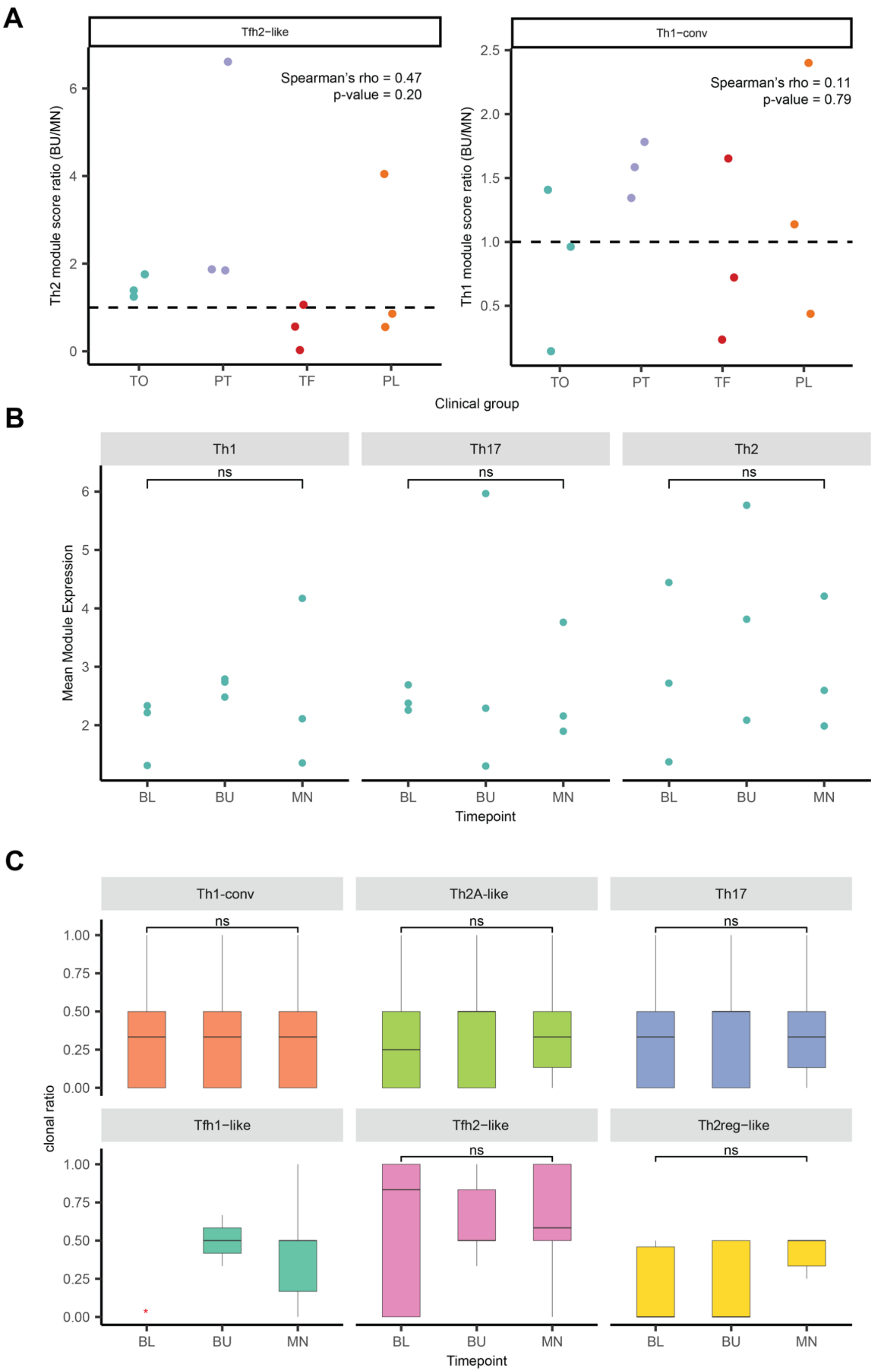

Supplementary Figure 13. OIT-induced changes in Th gene module expression by clinical group and in placebo patients. A, Degree of suppression in Tfh2-like and Th1-conv clonotypes by clinical group. Ratio of mean Th2 and Th1 module expression in Tfh2-like and Th1-conv clonotypes, respectively, from each patient was calculated between buildup (BU) and maintenance (MN) timepoints. Spearman's rho (n = 9) and p-value were derived from a Spearman correlation test between ratio and outcome within the treatment group (assigning TO as 2, PT as 1, and TF as 0). Dashed line indicates equal module expression between BU and MN (BU/MN ratio of 1). **B**, Mean expression of the Th1, Th2, and Th17 gene modules at baseline (BL), buildup (BU), and maintenance (MN) in Th1, Th2, and Th17 clonotypes from each of the three placebo patients. **C**, Fractional clonal expression over time of clones from placebo patients in each T helper subset. Fractional

clonal expression was defined as the fraction of cells within each clonotype that scored as module-expressing for the relevant gene module (Th1, Th2, or Th17) at a given time point. Clonotypes were only included at time points for which they had at least two cells recovered. Red ‘*’ indicates no clonotypes meeting the criteria were recovered. Paired (B) and unpaired (C) statistical significance was tested using Wilcox rank-sum test and adjusted using Bonferroni correction (**B**, **C**).

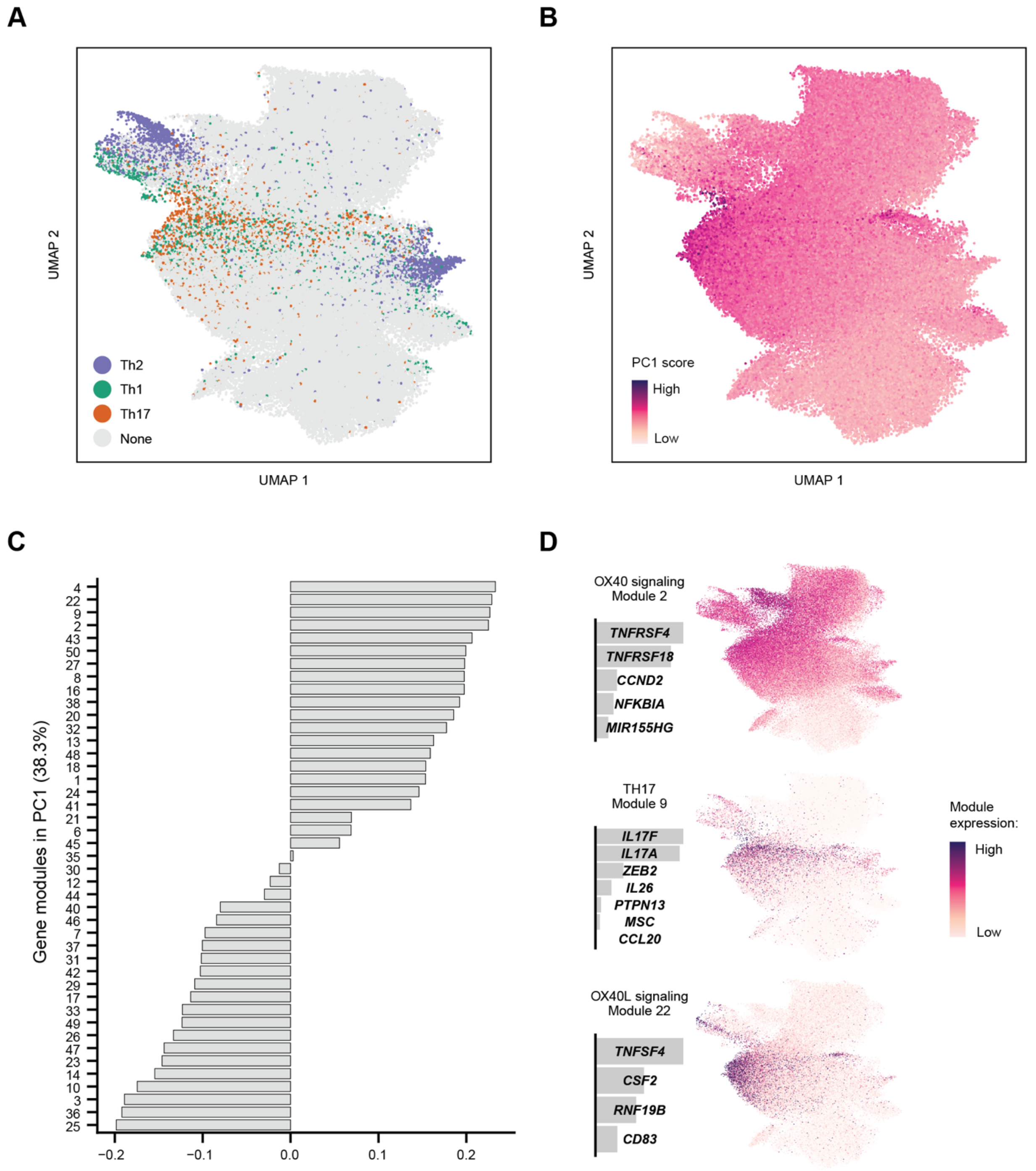

Supplementary Figure 14. Modules enriched in principal component 1 (PC1) at baseline. A principal components analysis was done using the 50 gene modules as features and all CD154+ cells at baseline as the input data. **A**, UMAP of all cells colored by Th module classification. Module expression was thresholded as described in **Methods**. **B**, UMAP of all cells colored by principal component 1 (PC1) score. **C**, Loadings of each gene module (labeled by number on the y-axis) in PC1. **D**, Gene loadings and module expression overlays on the UMAP coordinates, for selected modules positively enriched in PC1.

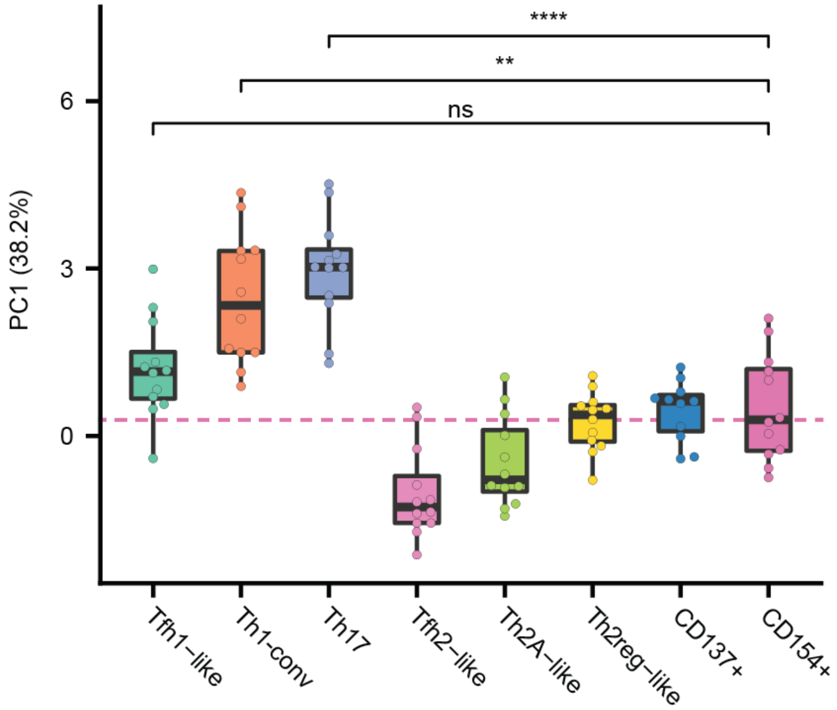

Supplementary Figure 15. Principal component 1 (PC1) scores of T cell subsets. Each data point represents the mean PC1 score (**Supplementary Figure** 14) of all cells of a given subset from a single patient at all timepoints. The dotted line represents the median average score of PC1 in the CD154+ subset. P-values were calculated using a paired two-sided Wilcoxon rank-sum test and were adjusted with Bonferroni correction. ‘*’ refers to an adjusted p-value of <0.05 by a Wilcoxon rank-sum test, “**” refers to adjusted p-value of <0.005, and “****” refers to adjusted p-value of <0.0005.

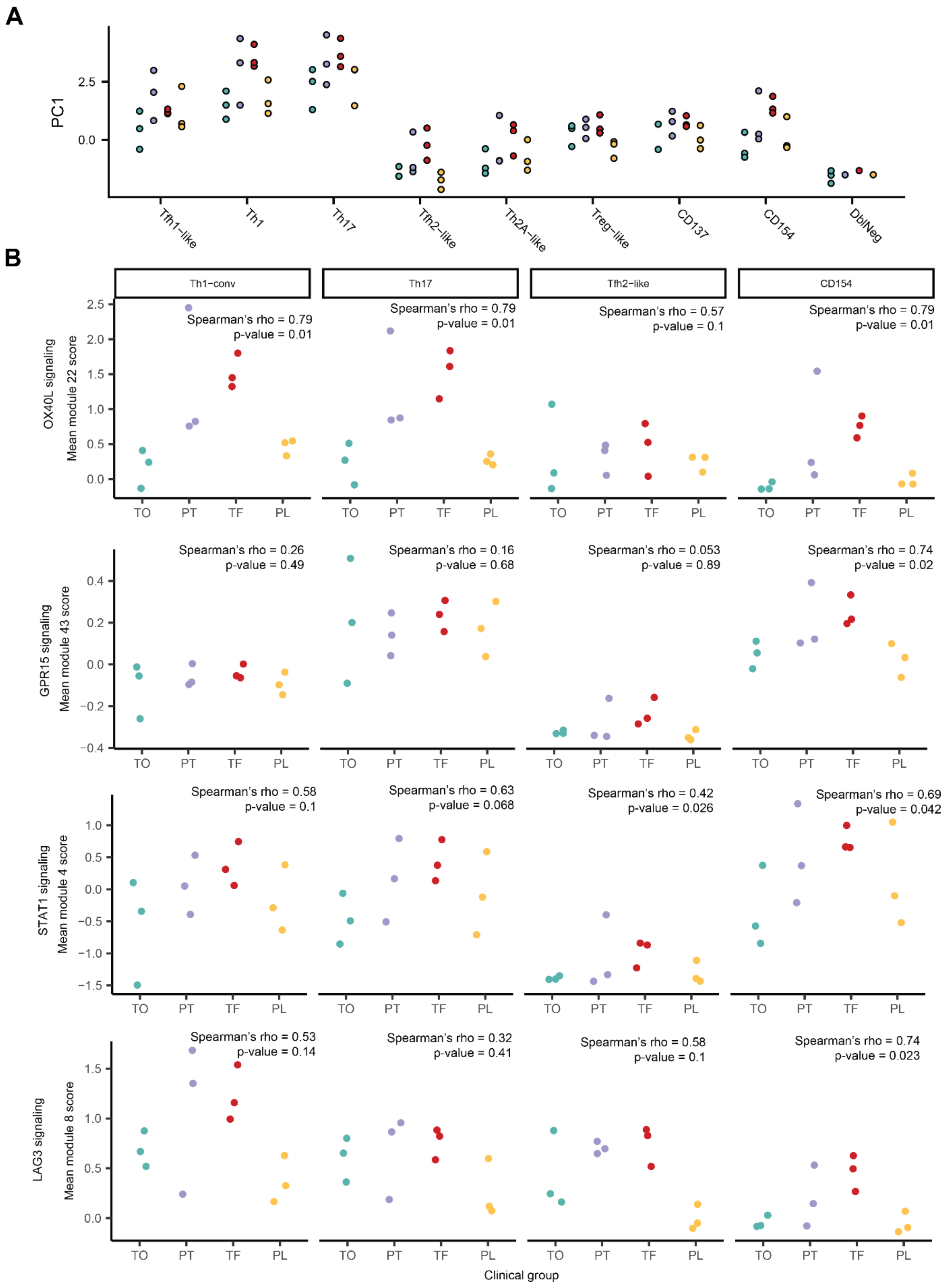

Supplementary Figure 16. PC1 scores and mean scores of modules enriched in PC1 by cell subset and treatment outcome. Module expression and PC1 scores of each Th subset shown were averaged by patient at all time points. "CD154" includes all recovered CD154+ cells not categorized as any of the six Th subsets shown in **Figure** 5. **A**, PC1 scores, as shown in **Figure** 5D, colored by treatment outcome and grouped by T cell subsets (green = TO, purple = PT, red = TF, yellow = PL). **B**, Mean module expression of indicated modules in Th1-conv, Th17, Th2A-like, and rest of CD154+ cells by patient, and colored by outcome. Spearman's rho (n = 9) and p-

value are from a Spearman correlation test between mean module scores and treatment outcome, as described in **Methods**.
